# Attrition in Digital Self-Management Interventions for Patients With Metabolic Dysfunction Associated Steatotic Liver Disease (MASLD): Mixed Methods Systematic Review

**DOI:** 10.2196/89124

**Published:** 2026-08-10

**Authors:** Rui Pang, Yihong Xu, Xiaoxiao Yu, Jianan Wang, Zhichao Yang, Haofen Li, Xiaojie Zhang, Ning Chen, Xiao Liang, Hongying Pan

**Affiliations:** 1 Department of Nursing, Sir Run Run Shaw Hospital, Zhejiang University School of Medicine, 3 Qingchun East Road, Hangzhou, Zhejiang, 310016, China, 86 13857188922; 2 Department of General Surgery, School of Medicine, Sir Run Run Shaw Hospital, Zhejiang University, Hangzhou, Zhejiang, China

**Keywords:** MASLD, digital health, attrition, retention, self-management, mixed method review, metabolic dysfunction associated steatotic liver disease, mobile phone

## Abstract

**Background:**

Lifestyle modification delivered through digital self-management is central to metabolic dysfunction-associated steatotic liver disease (MASLD) care, yet long-term engagement remains the threshold beyond which clinical benefit is realized. Understanding attrition requires examining both retention (dropout) and adherence (usage quality), which are often evaluated in isolation. Existing systematic reviews of digital interventions for MASLD have focused predominantly on clinical effectiveness, leaving less attention on attrition.

**Objective:**

This study aimed to integrate quantitative retention metrics with qualitative adherence insights and characterize the determinants of attrition in digital MASLD self-management interventions.

**Methods:**

Following PRISMA-S (Preferred Reporting Items for Systematic Reviews and Meta-Analyses literature search extension) and PRISMA (Preferred Reporting Items for Systematic Reviews and Meta-Analyses) 2020 guidelines, a comprehensive search of five databases (PubMed, Web of Science, Embase, Cochrane Library, and CINAHL) was conducted. The initial search was conducted in June 2025, and a subsequent update was made on April 17, 2026. Eligible studies enrolled adults with MASLD or nonalcoholic fatty liver disease in structured digital self-management interventions reporting retention or adherence data. Methodological quality was assessed using the Mixed Methods Appraisal Tool. A convergent segregated design was adopted. Retention proportions were pooled using random-effects meta-analysis with logit transformation, restricted maximum likelihood estimation, and Hartung-Knapp-Sidik-Jonkman adjustment. Adherence data were synthesized through inductive framework synthesis. Findings were subsequently integrated narratively.

**Results:**

In total, 21 studies met the eligibility criteria, of which 15 (n=1,032) contributed to the quantitative synthesis. The pooled retention proportion was 80% (95% CI 72%-87%) with substantial between-study heterogeneity (I²=73.6%). App-based platforms showed the highest point estimate and the lowest within-group heterogeneity, although no subgroup difference reached statistical significance. Adherence varied widely and was not amenable to meta-analytic pooling. Thematic synthesis identified 4 interacting domains shaping adherence, namely platform and design, human support and professional integration, motivational and behavioral strategies, and patient-level characteristics. Access friction at entry, gated coaching architecture, the absence of proximal biological feedback, and psychological comorbidity recurred as attenuators of long-term engagement.

**Conclusions:**

This review innovatively integrates retention and adherence to provide a comprehensive framework of attrition dynamics specific to MASLD. While retention compared favorably with adjacent fields, long-term adherence depended less on platform type than on accessible human support, alignment of feedback with the disease’s silent course, and psychological screening. Although evidence certainty was rated very low under Grading of Recommendations Assessment, Development and Evaluation, reflecting blinding constraints intrinsic to digital interventions and a predominance of pilot or feasibility designs, these findings carry clear real-world implications. Future interventions would benefit from establishing standardized, component-level reporting that distinguishes retention from adherence, to reliably evaluate the true therapeutic potential of digital MASLD interventions.

## Introduction

With an estimated global prevalence of 30%, the metabolic dysfunction-associated steatotic liver disease (MASLD) has already become the most concerning chronic liver diseases worldwide [1]. Generally, MASLD is primarily characterized by progressive intrahepatic fat accumulation, driven by a complex interplay of risk factors including gut microbiota alterations, unhealthy lifestyle behaviors, genetic susceptibility, and multimorbidity [2]. Increasingly high prevalence worsens the difficulty in management of patients with MASLD, contributing MASLD to the second leading causes of end-stage liver diseases [3,4]. Additionally, there are various extrahepatic complications associated with MASLD, such as obesity [1], cardiovascular disease [5], type 2 diabetes [5], and so on. Given that numerous adverse outcomes exist and pharmacological options are limited to specific patient subsets [6], lifestyle modification remains the cornerstone of therapy for all stages of MASLD [7]. Therefore, self-management interventions, which are designed to empower patients to adopt and maintain these crucial lifestyle changes, represent the primary therapeutic approach.

Self-management interventions are inherently complex, multicomponent processes that necessitate continuous patient engagement in behavioral goal-setting, self-monitoring, and education [8]. Delivering these interventions solely through the conventional approach of in-person office visits has proven challenging and resource-intensive, which may reduce the effectiveness of lifestyle interventions over time. In contrast, intelligent information technology circumvents geographical and temporal boundaries, thereby enhancing accessibility and providing a foundation for adaptive, individualized patient support [9]. Therefore, the delivery system is evolving toward a more flexible hybrid model that combines in-person visits, web-based visits, or both [7]. A systematic review showed that lifestyle modification based on eHealth technologies for patients with MASLD is significantly effective for clinical outcomes and biological markers, such as BMI, aspartate aminotransferase, and alanine aminotransferase [10]. However, despite this comparable efficacy, these emerging digital tools grapple with significant difficulties in achieving adequate patient uptake and maintaining long-term engagement, which critically limits their ultimate therapeutic potential.

Digital health care programs possess an inherent advantage over traditional trials in measuring engagement, as objective measures of intervention usage are readily available to researchers. In that, the conception of retention and adherence deserves more attention. Retention refers to the proportion of participants who remain enrolled and complete the study or intervention follow-up, reflecting trial-level feasibility and continuity [11]. In contrast, adherence denotes the extent to which participants engage with the intervention as intended, which is typically operationalized using metrics such as task completion, log-in frequency, or duration of use [12]. Unfortunately, existing systematic reviews of digital interventions for MASLD have focused predominantly on clinical effectiveness [10,13], reducing participant dropout to a mere denominator correction. Consequently, retention serves simply as a flow-diagram statistic for risk-of-bias appraisal, and adherence is typically evaluated in isolation. Crucially, the structural relationship between these 2 dimensions remains untheorized.

Notably, retention and adherence are inextricably linked constructs. While they share the common mechanism of diminishing interest, they represent distinct yet interconnected dimensions of the broader phenomenon of attrition. In his landmark “science of attrition,” Eysenbach [14] operationalized these dynamics as nonusage attrition (reflecting poor adherence) and dropout attrition (reflecting low retention). However, existing evidence remains fragmented, with most studies focusing on attrition as isolated outcomes [15-17]. When retention is more likely to be represented by quantitative analysis, adherence struggles with high heterogeneity, as Donkin et al [17] summarized various different methods for measuring adherence to electronic therapy (e-therapy). This core differences determine the difference in methodology selection. Although Jakob et al [16] proposed a customized scoring method to quantify adherence (calculated as the ratio of intended use to actual use) and examined determinants of engagement with mobile health (mHealth) apps among populations with noncommunicable diseases. But this method also ignored the possibility of different evaluation methods and dismissed the significance of retention. Moreover, the risk of “conceptual compression,” where the attempt to assign a single score oversimplifies the multifaceted nature of attrition, is the common disadvantages across a large body of studies [15,18].

Recently, attrition has progressively been elevated from a methodological footnote to a primary object of systematic synthesis. Linardon and Fuller-Tyszkiewicz [19] conducted a parallel synthesis across 70 randomized smartphone trials in mental health, quantifying both study attrition and intervention adherence and identifying enrollment modality, monetary compensation, and reminder use as significant moderators. To date, no systematic review has comprehensively evaluated attrition patterns specifically for digital MASLD self-management interventions, despite their growing integration into treatment. Attrition issues encompass both the measurable patterning of who stays and the interpretive complexity of why they do, therefore, a mixed methods systematic review offers the ideal framework. It uses quantitative synthesis to accurately map retention patterns, while implementing qualitative synthesis to unpack the heterogeneity of adherence without oversimplifying it into a single metric. Therefore, this mixed methods review aims to integrate quantitative retention metrics with qualitative adherence insights to evaluate digital MASLD self-management interventions. By uncovering the multifaceted barriers and facilitators to long-term engagement, this synthesis seeks to inform the design of more patient-centered programs.

## Methods

### Design

We used a result-based convergent approach to provide a comprehensive synthesis for different research types on the impact of retention and adherence. Simple meta and narrative synthesis was used for quantitative findings and thematic synthesis for qualitative findings. This review is reported in accord with the PRISMA (Preferred Reporting Items for Systematic Reviews and Meta-Analyses) and PRISMA-S (Preferred Reporting Items for Systematic Reviews and Meta-Analyses literature search extension) reporting guideline [20,21], and its protocol has been registered on PROSPERO before the process of data extraction (identification CRD420251068217).

### Search Strategy

We searched 5 electronic databases (PubMed, Web of Science, Embase, Cochrane, and CINAHL) in the last 10 years. The complete search strings for all databases, including Boolean operators, wildcards, MeSH, and applied filters, are provided in section S1 in Multimedia Appendix 1. To maximize the capture of relevant literature, we used a highly sensitive, 2-concept search strategy. This strategy combined (1) terms for metabolic dysfunction-associated steatotic liver disease (MASLD, metabolic dysfunction-associated fatty liver disease, or non-alcoholic fatty liver disease), and (2) terms for digital technologies (eg, eHealth, mHealth, telemedicine, mobile apps, wearables, and web-based interventions). Notably, we intentionally excluded “self-management” or “lifestyle” as a search concept. The rationale for this omission is that the terminology for such interventions (eg, “behavioral modification,” “patient education,” “coaching,” or “digital lifestyle support”) is highly heterogeneous and inconsistently indexed across bibliographic databases. Adding them to the electronic search string would risk prematurely filtering out eligible studies. The final search strategy was developed by the review team, and reference-list screening of included studies was also performed to identify additional records.

### Eligibility Criteria

Studies were screened for inclusion based on the Population, Intervention, Comparison, Outcomes, and Study Design framework. The detailed inclusion and exclusion criteria are summarized in Table 1.

**Table 1.** Eligibility criteria for study inclusion and exclusion.

Category	Inclusion criteria	Exclusion criteria
Population	Adults (≥18 y) diagnosed with MASLD^a^, MAFLD^b^, or NAFLD^c^.	Pediatric populations (<18 y) or patients without a diagnosis of MASLD, MAFLD, or NAFLD.
Intervention	Digital health interventions (eg, mobile apps, web-based programs, telehealth, wearables, or SMS) targeting self-management or lifestyle modification (diet, physical activity, weight, or psychological support).	Interventions focused exclusively on pharmacological or surgical treatments; digital tools used solely for data collection without interventional components.
Outcomes	At least one quantitative measure of retention (eg, completion rate) or qualitative or mixed-methods accounts of adherence barriers, facilitators, or engagement patterns.	Studies lacking specific data or insights into participant retention, adherence, or engagement behavior.
Study design	Original primary research articles.	Reviews, editorials, study protocols, conference abstracts, and nonprimary research.
Language	Full text available in English.	Non-English publications or studies where the full text could not be retrieved.

a MASLD: metabolic dysfunction associated steatotic liver disease.

b MAFLD: metabolic dysfunction-associated fatty liver disease.

c NAFLD: non-alcoholic fatty liver disease.

### Selection Process

All retrieved records were imported into EndNote 20.5 (Clarivate), and duplicates were removed. Titles and abstracts of the remaining records were then independently screened by the same 2 reviewers (RP and YX) against the eligibility criteria. Subsequently, full texts were retrieved for all potentially eligible records and assessed independently by the same 2 reviewers. Any discrepancies were resolved through discussion, and a third reviewer (JW) was consulted when consensus could not be reached. The entire process was documented to ensure transparency and reproducibility, and a PRISMA flow diagram was used to illustrate study selection.

### Data Collection Process and Data Extraction

Data were extracted independently by 2 reviewers (RP and JW) using a prepiloted, standardized extraction form. Discrepancies were resolved by discussion and, where necessary, by re-examination of the original source text. Missing or ambiguous data were handled by contacting corresponding authors. The following variables were extracted: (1) bibliographic details: first author, year, country, and journal; (2) study design; (3) participant characteristics: enrolled and analyzed sample size, mean age, sex distribution, and mean BMI; (4) intervention characteristics: digital delivery modality, duration (weeks), and interactivity level; (5) retention outcome: numbers enrolled and completing the intervention at the primary end point, and the derived proportion; and (6) adherence outcomes: operational definition (or notation that none was provided), metric type, and reported rate.

Each intervention was classified a priori as high-interactivity or low-interactivity by both reviewers independently. High-interactivity was defined as the presence of bidirectional, personalized communication between the participant and a human professional or peer. Interventions in which all participant-facing communication was unidirectional were classified as low-interactivity. Discrepancies were resolved by consensus.

When retention data reported at multiple time points, the retention rate at the end of the active intervention period was extracted as the primary data point, to maximize comparability across studies with heterogeneous postintervention follow-up designs. Additionally, several included studies were associated with more than 1 publication reporting data from the same intervention cohort. In such cases, all companion publications were retained and cross-referenced, with data extraction anchored to the publication providing the most complete reporting of variables relevant to this review. Data unavailable in the primary source were supplemented from companion publications, with the originating source noted in the extraction form. Studies sharing a participant cohort are identified in sections S2-S4 in Multimedia Appendix 1 with a shared superscript indicator; participant counts were deduplicated before meta-analysis to ensure the independence of observations.

### Risk-of-Bias Assessment

The methodological quality of all included studies was appraised using the Mixed Methods Appraisal Tool (version 2018) [22]. This validated instrument enables consistent evaluation across qualitative, quantitative (randomized controlled trial [RCT] and non-RCT), and mixed methods designs through 5 structured criteria specific to each study type. Each criterion is rated as “Yes,” “No,” or “Can’t tell,” with the overall quality score expressed as a percentage, where each unmet or unclear criterion results in a 20% reduction. For mixed methods studies, the final quality rating is determined by the weaker component between the qualitative and quantitative parts, while in multimethod studies, the mean value of both components is calculated. Studies were categorized as high (80%‐100%), moderate (40%‐79%), or low (0%‐39%) quality. The primary screening was undertaken by RP, and 20% (4/21) of the included studies were independently reassessed by 2 additional reviewers (YX and XY) to ensure consistency. Any discrepancies in scoring were discussed until consensus was achieved. No studies were excluded solely on the basis of methodological quality. A summary of the risk-of-bias assessment is presented in Table 2, and the full item-level Mixed Methods Appraisal Tool ratings are provided in section S5 in Multimedia Appendix 1.

**Table 2.** Risk of bias summary table.

Study type	Studies, n	High quality	Moderate quality	Low quality	Main methodological concern
Quantitative randomized trials	9	9	0	0	Outcome assessor blinding was not feasible or not reported in 5/9 studies.
Quantitative non-randomized studies	7	6	1	0	Confounding was not accounted for in 5/7 studies; one study had unclear representativeness.
Mixed methods studies	7	5	2	0	Two studies had limitations in interpretation of integrated outputs and adherence to component-specific quality criteria.
Overall	23	20	3	0	No study was excluded based on methodological quality.

### Statistical Analysis

All quantitative analyses were conducted in R (version 4.5.1; R Core Team) using the *metafor* package (version 4.6). Retention proportions were transformed to the logit scale before pooling to stabilize variance and normalize the sampling distribution. A random-effects model was fitted using the Hartung-Knapp-Sidik-Jonkman method [23] for variance estimation. This method allows the model to estimate the weighted pooled average with greater precision, also reducing the rate of false positives, particularly in meta-analyses with a small number of studies or substantial heterogeneity. The restricted maximum likelihood estimator was used to estimate the between-study variance component (τ²). Pooled estimates and 95% CIs were back-transformed to the proportion scale for reporting. Statistical heterogeneity was quantified using the Cochran Q test, the *I*² statistic, and the τ² estimate. The *I*² statistic describes the proportion of total variance attributable to between-study heterogeneity, but does not indicate the absolute magnitude of variation in true effects across populations [24]. Therefore, we calculated a 95% prediction interval for interpreting the weighted pooled average estimate of meta-analysis in the real-world.

Each prespecified variable (mean age, proportion of male participants, mean BMI, and intervention duration) was first tested in univariable models, followed by a multivariable model including conceptually relevant factors. The significance of moderators was assessed using the QM statistic, and the percentage of heterogeneity explained was quantified by *R*². Three preplanned subgroup analyses were conducted: (1) by digital delivery modality, (2) by study design, and (3) by intervention duration (section S7 in Multimedia Appendix 1). The robustness of the findings was examined using leave-one-out sensitivity analyses, and potential small-study effects were evaluated visually by funnel plot inspection and statistically using the Egger regression test. All statistical tests were 2-tailed, with a significance level of *P*<.05.

### Sensitivity Analyses

In total, 2 prespecified sensitivity analyses were performed to assess the robustness of the primary pooled retention estimate. First, to evaluate the influence of study design, the meta-analysis was repeated in the subset of randomized controlled trials only. Second, to evaluate the influence of the refined eligibility criteria applied during revision, the 4 previously meta-analyzed studies excluded under these refined criteria were reintroduced and the meta-analysis was refitted on the expanded datasets. Both analyses used the same inverse-variance random-effects model on the logit scale with restricted maximum likelihood estimation of τ², Hartung-Knapp adjustment, back-transformation to the proportion scale, and 95% prediction intervals. Robustness was judged by the consistency of the point estimate, the overlap of confidence and prediction intervals, and the stability of between-study heterogeneity (*I*² and τ²).

### Data Synthesis

We followed a convergent integrated mixed methods design (per JBI guidance), with quantitative and qualitative strands analyzed separately and then integratedly discussed in discussion.

#### Phase 1: Quantitative Synthesis

Retention data were meta-analyzed and reported as pooled proportions with 95% CIs and prediction intervals. Since adherence outcomes exhibited substantial heterogeneity in operational definitions and metrics, meta-analysis was pre-excluded.

#### Phase 2: Qualitative Synthesis

Text detailing barriers and facilitators of adherence was extracted verbatim. Moreover, 2 reviewers (RP and JW) independently applied framework synthesis, organizing extracted data within an explicit analytical structure through 3 stages: open descriptive coding of extracted text, focused coding through constant comparison across studies, and iterative grouping into analytical themes and organizing domains. Disagreements at any stage were resolved through discussion and consensus. This approach ensured interpretive transparency while maintaining an auditable analytical trail (the complete coding framework is in section S6 in Multimedia Appendix 1).

### Certainty of Evidence

The certainty of evidence for the primary outcome was assessed using the Grading of Recommendations Assessment, Development and Evaluation (GRADE) framework by 2 reviewers (HL and NC). Evidence was rated across 5 domains: risk of bias, inconsistency, indirectness, imprecision, and publication bias. The overall certainty was rated as high, moderate, low, or very low, and is reported in the Results section.

### Deviations From the Registered Protocol

Deviations from the registered protocol, including updated disease terminology (nonalcoholic fatty liver disease to MASLD), separation of retention and adherence as distinct outcomes, expansion of eligible digital platforms beyond smartphone apps, and change of statistical method from risk ratio to single-proportion meta-analysis, were determined before data analysis and are reported in full in section S8 in Multimedia Appendix 1.

## Results

### Study Selection

**Figure 1.**
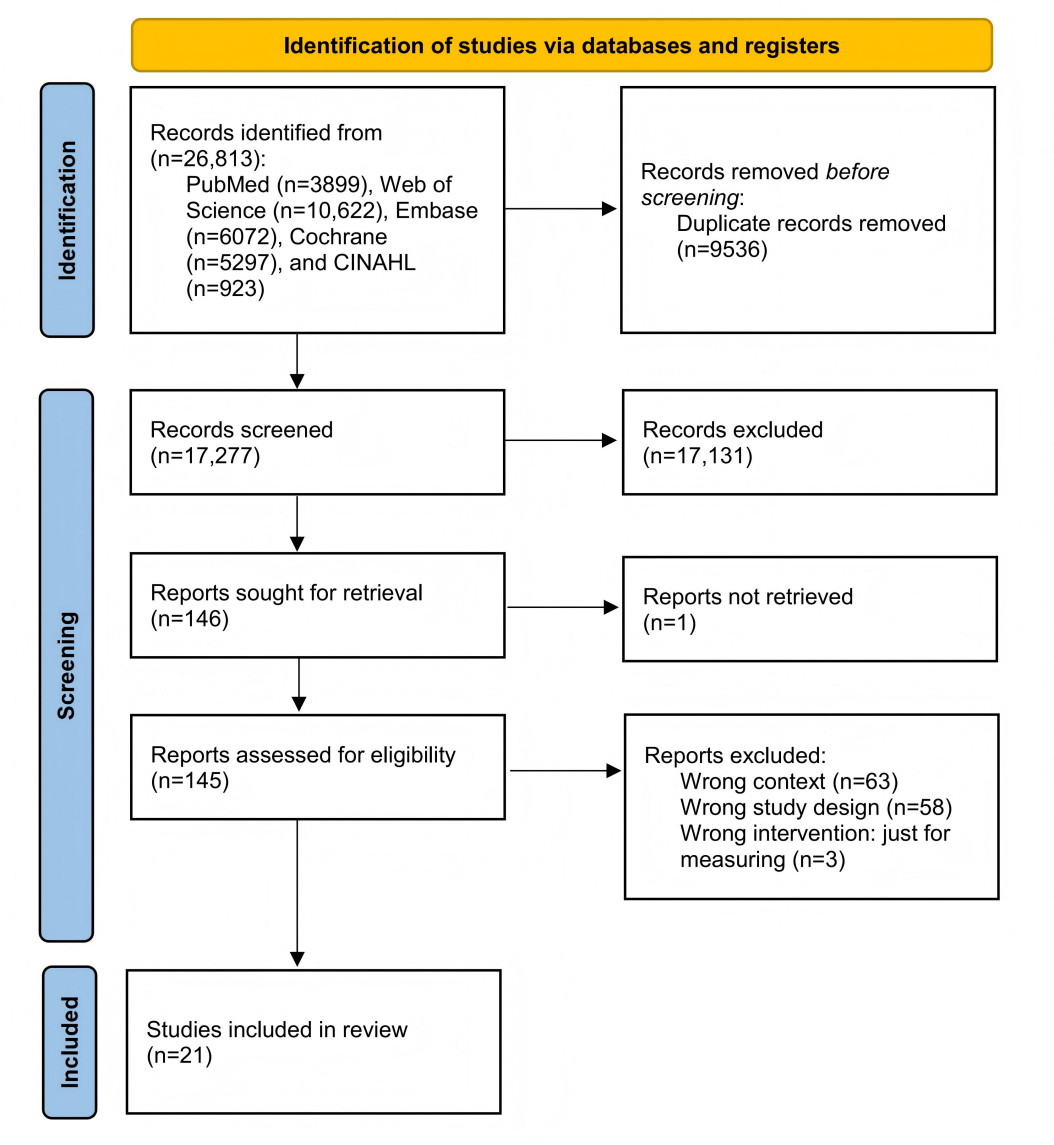
PRISMA (Preferred Reporting Items for Systematic Reviews and Meta-Analyses) flow diagram.

The first search was performed in June 2025 (22,864 citations) and was followed by an update on April 17, 2026 (3949 citations). Due to the broad search strategy prioritizing sensitivity, the initial screening yielded 17,131 records. At the title and abstract screening stage, the majority of records were excluded based on the following categories of reasons: (1) studies using digital tools solely as measurement or sequencing instruments (eg, digital calipers and next-generation sequencing platforms) without any intervention or management component, (2) studies not involving human participants with MASLD or nonalcoholic fatty liver disease, (3) nonprimary research (editorials, conference abstracts, and letters without data), and (4) studies unrelated to MASLD management. Given the volume of exclusions at this stage, individual record-level exclusion reasons were not tracked, consistent with established practice for large-scale systematic reviews. The remaining 146 studies were screened in full and excluded if applicable, leaving a final set of 21 studies [10,25-44]. The detailed process of study selection is presented in the PRISMA flow diagram (Figure 1).

### Study Characteristics

Our synthesis included 21 studies [10,25-44] published between 2016 and 2025. The geographic distribution of the evidence base was broad, yet heavily concentrated in high-income Western countries, with 3 studies [31,34,38] originating from the United States. Additional contributions came from Europe, including Germany (n=3) [29,43], Italy (n=2) [28,33], the United Kingdom (n=2) [25,40], and Iceland (n=2) [26,35]. Studies from Asia and Australia were also represented (Australia: n=2 [27,42], China: n=2 [36,41], Republic of Korea: n=2 [10,39], Thailand: n=1 [44], India: n=1 [30], and Singapore: n=1 [37]). Methodological approaches were notably heterogeneous. While RCTs (n=8) [10,31,36,37,39,41,43,44] represented a substantial portion of the included literature, the evidence base also comprised a large number of prospective single-arm, feasibility, or observational designs (n=9) [26,27,32-35,38,40,42]. A smaller subset of studies (n=4) [25,28-30] used mixed methods approaches to explore usability, acceptability, or specialist perspectives. Intervention sample sizes varied dramatically, from 22 to 314 participants. Participants were typically characterized by overweight or obesity, with mean ages ranging from 36.7 to 63.5 years across the included studies.

### Intervention Characteristics

The interventions leveraged various digital modalities to deliver the therapeutic content. Mobile apps (n=11) [10,25,26,30,35-41] were the most common platform, followed by telehealth-based delivery (n=4) [27,34,42,43], web-based programs (n=4) [28,29,32,33], and SMS text messaging interventions (n=2) [31,44]. Moreover, 1 entry was a qualitative study [25].

The primary therapeutic focus was most frequently on comprehensive lifestyle modification (n=11) [10,25-27,30,35,38-42,44] or weight loss (n=7) [28,31,33,34,36,37]. A smaller cohort of studies (n=3) [29,32,43] focused specifically on physical exercise. Intervention duration was highly variable, ranging from short-term 4-week protocols to long-term 24-month follow-up, with 12-week and 6-month being the most common. A key characteristic of most interventions was the integration of human support to facilitate a 2-way feedback loop, contrasting with 1 study [44] that used a purely 1-way information broadcast. This support was delivered by a range of professionals, including lifestyle coaches, dietitians, exercise physiologists, or advanced practice providers. Communication channels were diverse, encompassing asynchronous methods (eg, in-app chat, email, and bidirectional SMS text messaging) and synchronous modalities (eg, scheduled phone calls, group telehealth sessions, and real-time audio-visual supervision).

### Phase 1: Quantitative Synthesis

#### Study Selection and Characteristics

Of the 21 records retrieved, 15 independent studies [10,26,31-34,36-44] were included in the quantitative synthesis. Out of 15 studies, 5 [25,27-29,35] constituted companion publications reporting supplementary analyses or extended follow-up from the same intervention cohort as an already-included study, and were consolidated with their respective primary study for data extraction. One study (RESET program) [30] was excluded because its 3-arm design, allocating participants to interventions of differing content and intensity, precluded extraction of a single retention proportion comparable across arms without introducing conceptual heterogeneity.

Of the 15 included studies, 8 were RCTs [10,31,36,37,39,41,43,44] and 7 were observational studies [26,27,32-34,38,40]. In terms of delivery, most studies used mobile apps (n=8) [10,26,36-41], followed by telehealth (n=3) [34,42,43], SMS text messaging (n=2) [31,44], and web-based platforms (n=2) [32,33]. Interventions were categorized by duration into short-term (≤12 wk, n=6) [26,32,34,36,39,42] and long-term (≥24 wk, n=9) [10,31,33,37,38,40,41,43,44]. Participant mean age varied across the cohort, and BMI data were reported in 14 of the 15 included studies [10,26,31-34,36-39,41-44].

#### Primary Meta-Analysis of Retention

**Figure 2.**
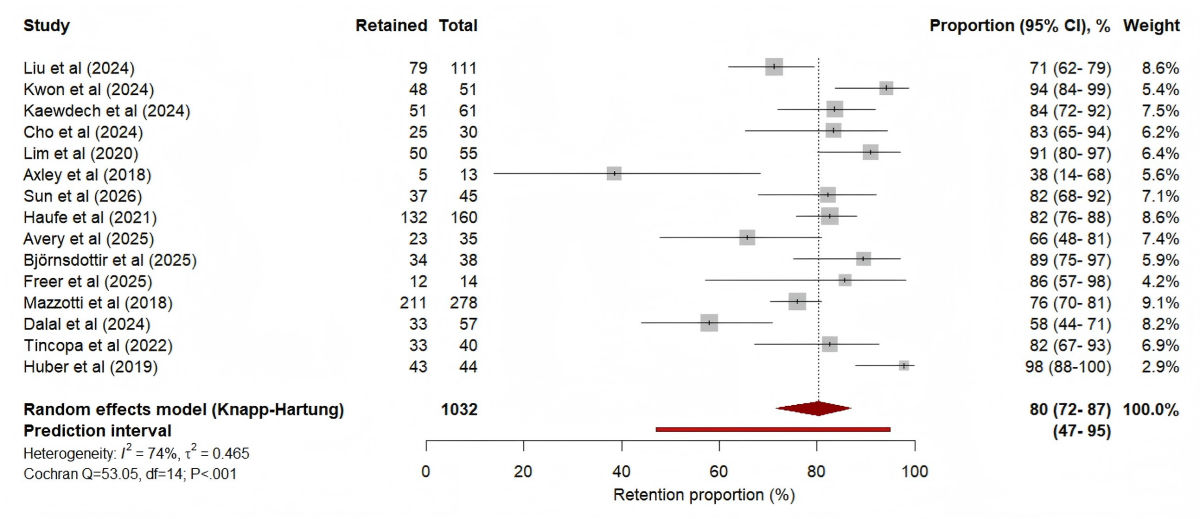
Forest plot of participant retention in digital self-management interventions for adults with MASLD. Data from 15 studies (8 RCTs, 7 observational; 2018‐2026) [10,26,31-34,36-44] were synthesized using a random-effects meta-analysis (Knapp-Hartung method). Square sizes reflect the relative statistical weight of each study, with horizontal lines indicating 95% CIs. The overall pooled average retention is represented by the bottom diamond, whose width corresponds to the 95% CI (precision of the mean effect). The bold red line denotes the 95% prediction interval, which represents the range within which the true retention proportion of a future comparable study would plausibly fall, thereby capturing between-study heterogeneity in addition to sampling error.

The pooled retention proportion across all 15 studies, estimated using a random-effects model with logit transformation, restricted maximum likelihood τ² estimation, and Hartung-Knapp-Sidik-Jonkman-adjusted CIs, was 80% (95% CI 72%‐87%). To characterize the distribution of true effects across different clinical and implementation contexts, the 95% prediction interval (PI) was calculated and extended from 47% to 95%. This wide PI indicates that although the average retention is high, the underlying true retention in a new comparable study could plausibly range from 47% to as high as 95%, reflecting substantial between-setting variability rather than statistical imprecision alone. Substantial between-study heterogeneity was observed (τ²=0.46, 95% CI 0.19‐2.08; *I*²=73.6%, 95% CI 56%‐84%; H=1.95; Q_14_=53.05; *P*<.001), underscoring the importance of the PI as a complement to the pooled estimate. Forest plots of individual study estimates and the overall pooled result are presented in Figure 2.

#### Assessment of Small-Study Effects

The funnel plot of logit-transformed retention proportions against SEs appeared broadly symmetric upon visual inspection, although 1 study [31] with a markedly lower retention and larger SE was positioned at the left tail (section S9 in Multimedia Appendix 1). Formal statistical testing did not indicate significant small-study effects: Egger linear regression test (2-tailed) yielded *t*_13_=1.50 (*P*=.16), and Begg rank correlation test (2-tailed) yielded *z*=1.24 (*P*=.22). These results suggest no statistically significant asymmetry in the funnel plot; however, given the limited number of included studies (n=15)[10,26,31-34,36-44], these tests have low statistical power to detect moderate asymmetry, and the possibility of small-study effects cannot be entirely excluded.

#### Result of Sensitivity Analysis

To assess the robustness of the primary pooled estimate to the inclusion of observational studies, a sensitivity analysis restricted to RCTs only (n=8; n=526 enrolled; n=427 retained) [10,31,36,37,39,41,43,44] was performed. The RCT-only pooled retention proportion was 81% (95% CI 67%‐90%; τ²=0.499, 95% CI 0.13‐3.56; *I*²=73.7%; Q_7_=26.60; *P*<.001), which was virtually identical to the primary pooled estimate of 80%. The PI for the RCT-restricted analysis ranged from 42% to 96%. The consistency between the primary and sensitivity analyses supports the validity of combining RCTs and observational studies in the primary synthesis, although substantial within-design heterogeneity persisted in the RCT-only model, suggesting that study design alone does not explain the observed variability in retention rates. A prespecified sensitivity analysis reintroducing 4 previously meta-analyzed studies excluded under refined eligibility criteria yielded a pooled retention of 82.5% (95% CI 75%‐88%; 95% PI 49%‐96%; *I*²=75.6%; n=19) [10,26,31-34,36-48], virtually identical to the main estimate, supporting the robustness of the primary analysis (supplementary file S11 and Table S11.2 in Multimedia Appendix 1).

#### GRADE Assessment

The certainty of evidence for participant retention in digital MASLD interventions was assessed using the GRADE framework, which is presented in Table 3. The overall certainty was rated as very low (⊕◯◯◯), reflecting 2 independent grounds for downgrading from the “low” starting point established by the inclusion of observational studies. First, risk of bias was rated as serious. Among the 8 RCTs [10,31,36,37,39,41,43,44], blinding of participants and intervention providers was not feasible given the nature of digital self-management tools—a limitation inherent to the intervention type rather than specific to individual study designs. Among the 7 observational studies [26,32-34,38,40,42], all were single-arm designs without control groups, restricting causal inference. Second, inconsistency was rated as serious, as substantial between-study heterogeneity was detected (*I*²=73.6%, 95% CI 56%‐84%; τ²=0.4650; Q_14_=53.05; *P*<.001), and the PI (47.01%‐94.98%) demonstrated that the true retention rate could plausibly range from below 50% to nearly 95% in future comparable settings. Univariate meta-regression of mean participant age, BMI, and sex distribution did not account for this variability (all *R*²≤6.97%), indicating that the sources of heterogeneity remain unidentified in the current evidence base. Consequently, the resulting “very low” certainty means that the pooled estimate should be regarded as a provisional summary of an average tendency, not as a precise or generalizable benchmark.

**Table 3.** Certainty of evidence for participant retention in digital MASLD^a^ interventions.

Outcome	Studies and participants	Pooled estimate	Prediction interval	Main limitations	Certainty
Retention rate in digital MASLD interventions	15 studies; n=1032	80.38% (95% CI 71.54%‐86.98%)	47.01%‐94.98%	Serious risk of bias^b^; serious inconsistency^c^; no serious indirectness^d^, imprecision^e^, or detected publication bias^f^	⊕◯◯◯ Very low

a MASLD: metabolic dysfunction-associated steatotic liver disease

b Risk of bias: Among the 8 RCTs, blinding of participants and intervention providers was not feasible given the nature of digital interventions, raising concerns in MMAT criteria Q2 and Q3. Observational studies (k=7) were single-arm designs without control groups, limiting causal inference. Downgraded one level (Serious).

c Inconsistency: Substantial between-study heterogeneity was detected (I²=73.6%, 95% CI 56.0%-84.2%; τ²=0.4650; Q=53.05, *df*=14, *P*<.001). The prediction interval (47.01%-94.98%) indicates that in a new comparable setting, true retention could plausibly range from below 50% to nearly 95%. Univariate meta-regression of age, BMI, and sex distribution did not explain this variability (all R²≤6.97%). Downgraded one level (Serious).

d Indirectness: The population (adults with MASLD), intervention (digital self-management tools), and outcome (participant retention) directly correspond to the research question. No meaningful indirectness identified. Not downgraded.

e Imprecision: The 95% confidence interval (71.54%-86.98%) is acceptably narrow given the sample size (n=1032). Although the prediction interval is wide (reflecting true heterogeneity rather than imprecision per se), the pooled estimate itself is based on sufficient events (816/1032). Not downgraded.

f Small-study effects (publication bias): Egger linear regression test showed no significant asymmetry (t=1.50, *df*=13, *P*=.16). The Begg rank correlation test was similarly nonsignificant (z=1.24, *P*=.22). Not downgraded.

### Phase 2: Qualitative Synthesis

#### Retention Rate

Where formally reported, program retention ranged from 62% [31] to 93% [32], with the majority of studies exceeding 80%. Longitudinal attrition was most clearly characterized in the web-based lifestyle modification program followed over 5 years [33], in which retention declined from 76% at 6 months to 43% at 24 months, before stabilizing after year 3. Older age (odds ratio [OR] 0.76, 95% CI 61%-91%, per decade) and comorbid diabetes (OR 0.33, 95% CI 22%-49%) were independently associated with lower attrition in this cohort, suggesting that perceived clinical necessity may function as a retention-promoting factor across extended follow-up periods [28]. Similarly, in the Behavioral Resources and Intervention through Digital Group Education telehealth program [34], older participants and those with cirrhosis demonstrated significantly higher session completion, consistent with this pattern.

Attrition patterns were rarely explained in sufficient clinical detail. Where dropout reasons were documented, personal illness, occupational commitments, and family obligations predominated, with loss of interest cited in a minority [35]. In the largest trial, the majority of dropouts were attributable to COVID-19 restrictions rather than disengagement, complicating interpretation [36]. Notably, several studies were unable to contact dropouts for follow-up, whether due to regulatory constraints or loss to follow-up, leaving the mechanisms of late attrition largely uncharacterized. The predominance of situational rather than motivational explanations for withdrawal, where data existed, suggests that structural barriers may account for a greater share of retention failure than is typically acknowledged in digital health trial reporting.

#### Adherence

##### Measurement Heterogeneity and Reporting Practices

Substantial variation was observed in the conceptualization, operationalization, and reporting of adherence across studies. Findings are therefore presented as a narrative synthesis and precluded meta-analytic pooling.

Commonly reported indicators included platform engagement metrics, intervention completion rates, and self-monitoring behaviors, particularly dietary and physical activity tracking. Additional measures comprised predefined adherence thresholds, wearable-device use, automated communication responsiveness, and usability assessments (Table 4). Notably, adherence was increasingly assessed in relation to specific behavioral components, such as exercise participation or dietary self-monitoring, rather than overall intervention exposure alone, although most of these metrics relied on self-reported data.

**Table 4.** Methods for measuring adherence to digital health interventions as reported by included studies^a^.

Measure of adherence	Example	Frequency of reporting
Platform engagement metrics	login frequency, platform access, module views, login duration, account activation	9
Intervention completion and participation	session, module, and program completion, coaching attendance, consultation participation	8
Self-monitoring behaviors	food diary, meal logging, food photo upload, weight logging, progress report submission, goal-setting tool use	8
Predefined adherence thresholds	≥66%‐80% prescribed sessions, intake, or activities completed	5
Physical activity adherence	daily steps, activity duration/intensity, valid wear days, wearable device use	5
Automated communication responsiveness	SMS or chatbot response rate	2
Usability or user experience assessment	MAUQ^b^ score	1

a Of the 21 studies included in this review, 18 (85.7%) reported adherence or engagement data in some form. Several studies employed more than one measure; the total therefore exceeds 18. Studies reporting no formal adherence data: Cho 2024 [39], Mazzotti 2018 [33], Petroni 2024 [28], and Kaewdech 2024 [44].

b MAUQ: Mobile App Rating Scale User Version

##### Adherence Rates

Adherence to digital interventions demonstrated substantial variation across platforms and behavioral components (Table 5). Smartphone app–based interventions generally achieved relatively high initial adherence to dietary self-monitoring (eg, 82.6% in SMART-Liver [Doing lab]), yet adherence differed considerably across tasks, with participants in nBuddy (Verita Analytics) showing greater compliance with weight logging (77%) than meal logging (56.7%) [37]. Adherence also declined consistently over time: in the Sidekick program [35], active engagement decreased from 6.8 days per week during the intensive phase to 2.38 days per week during maintenance, while response rates in SMS-based interventions [31] fell from 70% in week 1 to approximately 40% thereafter. By contrast, wearable-based interventions demonstrated very high technical adherence [38], although this did not necessarily translate into meaningful behavioral adherence or lifestyle modification.

**Table 5.** Adherence measurement methods, reported rates, and key findings across included studies of digital self-management interventions for metabolic dysfunction-associated steatotic liver disease (n=21^a^).

Project and platform	Study	Design	Adherence measures	Key adherence finding
Smartphone app–based interventions (n=11)				
SMART-Liver app	Kwon 2024 (South Korea, n=111, 6 mo) [10]	RCT^b^	Diet-log compliance rate (intended: 3 times/d)	Mean diet-log compliance: 82.6% (SD 12.9%) at 3 months; 79.8% (SD 13.9%) at 6 months. Participants with ≥90% compliance showed significantly greater improvements in weight, BMI, liver fat score, AST^c^, and γ-GT.
nBuddy app	Lim 2020 (Singapore, n=55, 6 mo) [37]	RCT (pilot)	Login frequency; meal logging rate (daily); weight logging rate (twice weekly); automated step count	76% of completers logged in on >75.3% of 182 days. Mean login rate: 87.6% (SD 19.6%) in months 1‐3, declining to 78.1% (SD 28.2) in months 4‐6. Meal logging: 56.7% (SD 51.6%) of recommended frequency; weight logging: 77.0% (SD 28.5%). Dropout: 5% (5/55).
Dr. Coach app	Cho 2024 (South Korea, n=60, 4 wk) [39]	RCT	No formal app usage frequency data reported	—^d^
Sidekick app	Björnsdottir 2025a (Iceland, n=38, 12 weeks, active phase) [35]	Feasibility study	Proportion active each week throughout; daily missions completed; active days/week; app usability (MAUQ^e^, 0‐7 scale); high engagement defined as ≥5 active days/week	High engagement: 22/38 (58%). Mean active days: 6.8/week (IQR 4.6‐7.0). Mean MAUQ: 6.3/7 (IQR 5.8‐6.7). Coaches sent mean 23.5 messages (SD 10.3); participants sent mean 15.5 (SD 12.4); mean coach response time 1.2 days.
	Björnsdottir 2025b (Iceland, n=38, 6-month maintenance phase) [26]	Feasibility study	Active weeks during maintenance (≥18/24 wk); active days/week; daily missions completed	28/34 (82%) who completed the active phase attended the 9-month visit. In the final maintenance week, 19/38 (50%) remained active in-app. Active for ≥5 days across ≥18/24 maintenance weeks: 17/38 (45%). Median active days/week: 2.38 (IQR 0.36‐6.08), compared with 6.8 during the active phase. Highly engaged maintenance participants showed significantly greater clinical improvements than less engaged participants.
MyTatva app + IoT^f^ devices (BCA + smartwatch)^g^	Soni 2025 (India, n=27, 12 wk) [30]	observatory	90-day program completion rate; food diary adherence (app-tracked); 15-day monitoring compliance	Program completion: 22/27 (81.5%). Food diary adherence was not reported quantitatively. Group C (diet + exercise+CBT^h^) achieved weight reduction in 100% of participants vs 85.7% (Group A) and 77.8% (Group B).
NUTRIEASE smartphone app + in-person monitoring	Liu 2024 (NUTRIEASE) (China, n=226, 12 wk) [36]	RCT	Food photo diary compliance (3 d per 4-wk period; assessed by dietitian review)	Food photo log compliance rate was not formally quantified as a percentage.
Changing Health web app	Hallsworth 2021 (VITALISE pilot) (UK, n=16, 12 wk) [25]	Pilot feasibility	Login frequency; module views (33 subsections); goal-setting and monitoring tool access; coaching sessions attended	Logged in at least once: 11/16 (69%). Mean logins: 3.6 (SD 3.5; range 0‐14). Goal-setting and monitoring tools accessed 22 times total (mean 4 per tool). Coaching accessed: 3/11 (27%). Most activity occurred in the first 4 weeks; minimal engagement thereafter.
	Avery 2025 (VITALISE feasibility) (UK, n=35, 6 mo) [40]	Clinical feasibility	Account activation rate; login frequency; coaching session attendance; module completion	Account activation: 83% (29/35). Mean logins: 7 over 6 months (SD 11; median 3). Coaching attended (≥1 session): 8/35 (23%); mean sessions attended among those who accessed coaching: 4 (range 1‐6). Engagement concentrated in month 1.
Fitbit Zip + email feedback	Tincopa 2022 (USA, n=33, 6 mo) [38]	Pilot cohort	% of days with valid Fitbit wear data (≥300 steps); step trajectory pattern over 6 months	Median valid Fitbit wear days: 91% (completers). No significant overall step count change from baseline (*P*=.52). Step patterns: 4 increased and maintained, 19 maintained, 8 increased then returned to baseline.
WeChat mini-program (TLC)	Sun 2026 (China, n=89, 24 wk) [41]	RCT	Daily check-in completion; food photo upload frequency; heart rate armband usage	Per-protocol completion: 73/89 (82%). Intervention loss to follow-up: 8/45 (18%; 5 stopped intervention, 3 personal reasons); control: 8/44 (18%). Diet quality scores significantly improved in the intervention group (median difference 6 points; *P*=.04). No significant between-group difference in MET-minutes/week^i^ (*P*=.92).
Web-based platforms and exercise programs (n=4)				
Docebo e-learning web platform	Mazzotti 2018 [33] (Italy, n=679, 24 wk)	Observatory	No adherence data reported	—
	Petroni 2024 [28]	Longitudinal (5-y)		
Web-based exercise platform	Pfirrmann 2019 (Germany, n=43, 8 wk) [29]	Prospective study	≥80% recommended endurance session completion (primary acceptance metric); login frequency and duration; weekly progress report submission	74% (32/43) completed ≥80% of recommended endurance sessions. Total logins: 557 (mean 13.0, SD 8.0; range 3‐38). Total login duration: 6,548 min (mean 152.3 min, SD 93.8). Exercises completed: 1169 (730 endurance, 439 strength); 207 canceled; 72 additional spontaneous exercises. Progress reports submitted: 165 (mean 3.8/person; SD unreported). Reminders sent: 120 (mean 2.8/person). Login activity highest in weeks 1‐2, declining thereafter. 1 participant lost to follow-up (2.3%).
	Huber 2019 (Germany, n=44, 8 wk+20 wk follow-up) [32]	Prospective study	≥70% prescribed session completion; independent exercise continuation at 20-week follow-up	41/44 (93.2%) met the ≥70% session completion threshold. 63.4% continued regular independent exercise at the 20-week unsupervised follow-up. 2/44 did not meet the completion threshold.
Telehealth, group video conferencing, and hybrid programs (n=4)				
TeleHab app	Freer 2024 & Freer 2025 [27,42] (Australia, n=28, 12 wk)	Feasibility RCT + mixed methods	Exercise adherence (sessions completed/prescribed; threshold ≥66%); protein adherence (≥80% of recommended intake)	Exercise adherence (all 14 Tele-ProEx completers): mean 52 ± 36% — below the pre-specified≥66% feasibility threshold. Among 9 full completers: 73 ± 26%. Plant protein adherence (≥2 serves/d): 32%. Animal protein adherence (7‐10.5 serves/wk): 42%. Only 14% (n=2) met both protein targets simultaneously. Consultation attendance: 100% at week 1, 86% at week 2, 64‐71% at week 6.
BRIDGE (Zoom group telehealth)	Dalal 2024 (USA, 12 wk) [34]	Feasibility study	Session attendance rate; proportion completing all 6 sessions; enrollment rate	Enrollment rate: 57/119 (47.9%). All 6 sessions completed: 33/57 (58%). ≥5 sessions attended: 43/57 (75%). ≥1 session missed: 24/57 (42%). Older participants significantly more likely to complete all sessions (median age 61 vs 53.5 y, *P*=.01). Cirrhosis patients had the highest completion rate (83%). Most frequent reasons for absence: personal illness, family commitments, and work obligations (63% of missed sessions categorized as ’other/unknown’).
Garmin Forerunner 35+ online platform	Haufe 2021 (Germany, n=160 exercise group, 6 mo) [43]	RCT	Activity duration and intensity compliance; daily steps; % achieving ≥150 min/week aerobic activity	Mean weekly activity: 147±46 min (target: 150 min); mean steps/d: 9,612±2498. Exercise group dropout: 28/160 (17.5%).
SMS, social media, and messaging platform interventions (n=2)				
CareMessage SMS platform	Axley 2018 (USA, n=13 intervention arm, 22 wk) [31]	RCT (pilot)	Response rate to bidirectional SMS messages; 6-month follow-up completion	Follow-up completion: 8/13 (62%). Week 1 message response rate: 70%. Month 1: 56%. Months 2‐6: 41%‐45%. Two participants opted out of the program.
LINE Official Account (social media broadcast)	Kaewdech 2024 (Thailand, n=122, 24 wk) [44]	RCT	Content engagement (no tracking mechanism available)	Content engagement was structurally unverifiable — the LINE Official Account platform does not support read-receipt or access tracking. Dietary adherence was likewise unverifiable, as no validated Thai dietary assessment tool was available.

a Note. Adherence data are presented as reported by study authors. Where studies used pre-specified completion thresholds, these are stated in the Adherence Measure column.

b RCT: randomized controlled trial.

c AST: aspartate aminotransferase.

d Not applicable.

e MAUQ: Mobile App Rating Scale User Version.

f IoT: Internet of Things.

g BCA: body composition analyser.

h CBT: cognitive behavioral therapy.

i MET: metabolic equivalent of task

Importantly, several studies conflated retention (ie, completion of study procedures) with adherence (ie, fidelity to intervention behaviors). Although program completion rates frequently exceeded 80%, actual behavioral adherence was often either unmeasured or substantially lower, as illustrated by the Tele-ProEx study [27,42], in which only 14% (2/14) of participants achieved prescribed nutritional targets. This conceptual ambiguity obscures the phenomenon of participants remaining enrolled but behaviorally inactive, thereby limiting accurate evaluation of dose-response relationships and constraining deeper understanding of intervention failure mechanisms in digital health research.

### Qualitative Synthesis of Facilitators and Barriers

#### Overview

A total of four thematic domains were inductively identified: (1) platform and design, (2) human support and professional integration, (3) motivational and behavioral strategies, and (4) patient-level characteristics. Detailed facilitators and barriers within each domain are presented in Table 6.

**Table 6.** Qualitative synthesis of facilitators and barriers to adherence to digital self-management interventions for MASLD, organized by thematic domain^a^.

Domain and theme	Facilitators	Barriers
Platform and design		
Usability and accessibility	Integration into existing platform ecosystems eliminated download barriers and leveraged pre-established user familiarity. App-format delivery was reported as superior to web-link access. Passive data collection via wearables or AI-assisted logging substantially reduced manual burden.	Web-link delivery reduced motivation relative to app-format access. Device connectivity issues limited engagement. Restricting the platform to a single operating system (Android only) reduced potential reach. Primary login failure caused durable disengagement that was not reversed by subsequent outreach.
Content structure	Sequential (tunneled) module navigation was associated with greater progression through content. Diverse daily mission content maintained engagement variety over time. Audiovisual instruction formats supported exercise self-efficacy.	Excessive information volume was described as cognitively burdensome and contributed to early dropout. Content exhaustion, the absence of new material once existing modules were completed, was identified as a distinct barrier to sustained use. Lack of variety in app content was cited after the active coaching phase ended. Exercise platform inflexibility (inability to pause or rewind videos) impeded exercise completion.
Automation	Automated reminders (daily or weekly) were associated with maintenance of login habits. AI-powered food photo recognition reduced dietary logging burden. Passive IoT^b^ data capture via wearable devices ensured continuous objective monitoring without requiring active user effort.	Response rates declined substantially over the study period. No consensus was identified regarding the optimal frequency or intensity of automated contact to sustain engagement without inducing disengagement.
Human support and professional integration		
Professional coaching	Scheduled coach contact at defined intervals established personal accountability. Coach responsiveness within 24 hours was explicitly cited as a facilitator. Early rapport-building sessions in weeks 1‐2 were associated with sustained engagement throughout the active phase. Expert prescription adjustments based on participant-reported feedback enabled safe and progressive progression.	Low coaching uptake was observed despite availability. A structural barrier, requiring completion of all educational modules before coaching became accessible, was identified as a proximal cause of low uptake.
Group and peer support	Group telehealth formats (shared medical appointments) reduced stigma and built community accountability. Peer support channels within app platforms reinforced commitment to lifestyle modification. Optional bi-weekly group exercise sessions supported social accountability. Spanish-language group sessions reduced linguistic barriers and supported participation among Hispanic patients.	Group formats provided no inter-session digital contact or monitoring between sessions. Social comparison features within apps risk negative effects when other participants show low engagement.
Personalized feedback	Individualized prescriptions developed from baseline assessments (cardiopulmonary testing, dietary recall, activity monitoring) were associated with higher acceptability and safety. Bidirectional messaging enabled real-time tailoring of content to individually reported barriers and goals. Brief 1-on-1 breakout rooms within group sessions delivered individualized goal discussions in the context of a group program.	One-way broadcast content without personalized response (LINE Official Account) did not allow adaptation to individual needs. Highly individualized modules demanded substantial manual professional input, limiting the scalability of the intervention.
Motivational and behavioral strategies		
Initial motivation and disease context	Delivery at the point of MASLD^c^ diagnosis capitalized on heightened receptiveness to behavior change ('teachable moment’). Self-efficacy and perceived health benefits were identified as the key psychological mechanisms sustaining adherence beyond initial engagement. Early positive clinical outcomes (eg, visible weight loss or improved liver markers) reinforced continued engagement.	Many patients were unaware that lifestyle modification could meaningfully improve liver disease outcomes, reflecting a gap in disease-specific health literacy that attenuated initial motivation. Perception of the app as an unwanted reminder of illness was identified in some participants as a barrier to re-engagement.
Goal setting and behavioral monitoring	Progressive goal setting calibrated to individual baseline capacity and exercise intensity derived from cardiopulmonary testing was consistently associated with higher adherence. SMART^d^ goal frameworks with structured accountability tracking supported sustained engagement. Motivational interviewing was integrated in several programs and reported to enhance autonomous motivation. Completion incentives (eg, free transient elastography) were associated with higher retention.	No consensus was identified on the optimal frequency or intensity of goal-related check-ins or accountability contacts. Postintervention follow-up without active supervision was associated with relapse in physical activity and engagement.
Patient-level characteristics		
Demographics and clinical profile	Older age was associated with significantly higher session completion in the BRIDGE^e^ program and with lower attrition in the long-term web-based program. Comorbid diabetes was associated with significantly lower attrition (OR 0.33). Structured onboarding support (including digital literacy training) helped older patients overcome technology barriers.	Multimorbidity caused MASLD-related self-management to be deprioritized relative to other health conditions. Comorbid mental health conditions, particularly depression and anxiety, were consistently associated with lower adherence across multiple studies. Musculoskeletal pain and fatigue limited physical activity adherence.
Socioeconomic and structural factors	Telehealth delivery eliminated transportation barriers and supported participation among patients living in rural or remote areas. Insurance coverage removed out-of-pocket cost barriers to participation. Financial support was identified as an important enabler for lower socioeconomic participants.	Commercial insurance coverage did not always include program costs, creating participation barriers for some patients. Occupational commitments and shift-work schedules reduced available engagement time. Technology access gaps, including lack of internet connectivity or digital devices, were reported as enrollment barriers.
Cultural and dietary factors	Dietary alternatives tailored to local cultural food norms improved adherence and acceptability. Language-appropriate program content (Spanish-language sessions) removed a significant access barrier. Leveraging familiar digital ecosystems aligned with local communication culture (LINE in Thailand, WeChat in China) enhanced uptake.	Cultural unfamiliarity with recommended foods (plant-based protein sources including tofu and tempeh) substantially limited dietary adherence targets, despite high exercise satisfaction. Validated dietary assessment tools were absent for some study populations, making dietary adherence unverifiable. Women and Hispanic participants were disproportionately burdened by family and community obligations that competed with intervention engagement.

a Note. Factors are classified as either intervention-related (modifiable through programme design) or patient-related (largely fixed characteristics) in accordance with the framework proposed by Jakob et al, 2022 [16]. Entries without citations reflect consistent patterns observed across multiple studies without a single attributable source.

b IoT: Internet of Things

c MASLD: metabolic dysfunction-associated steatotic liver disease

d SMART: specific, measurable, achievable, relevant, and time-bound

e BRIDGE: Behavioral Resources and Intervention through Digital Group Education

#### Platform and Design Characteristics

The most consistent finding across design-related themes was that access failure at the entry point constituted the primary threat to participation [25,40]. For instance, the extra steps for gaining specific access to websites and applications are still viewed as a digital burden for end users [35]. Even worse, the failure in primary log-in largely led to the disengagement of participants, reducing the confidence and intention for behavior change [25], producing a ceiling effect whereby the most resource-intensive support features, typically positioned later in sequential program architectures. Automation mitigated some of this burden, yet declining SMS response rates over time suggest that automated accountability cannot substitute for the adaptive responsiveness that human contact provides [31]. Similarly, the tension between content depth and cognitive load remained unresolved: tunneled formats improved progression but risked early dropout, while open-browsing formats preserved autonomy at the cost of structure and supervision [25,35]. No design configuration has yet demonstrated long-term engagement across the full intervention trajectory.

#### Human Support and Professional Integration

Human support functioned as a necessary but insufficiently accessed component of most programs. Where coaching was actively received, its benefits—personalized prescription, early rapport, real-time adaptation—were consistently facilitative; yet uptake remained low, in part due to program architectures that made coaching contingent on previous content completion [25,40]. This creates an inherent inequity: participants with the lowest engagement received the least support precisely when they needed it most. Peer and group formats partially compensated for limited professional contact by providing social accountability and reducing stigma [34,37], yet their intersession reach was structurally limited. Participants indicated that the community attribute can significantly reinforce their commitment to lifestyle modifications [38]. Peer support or professional guidance in social groups can strengthen the sense of connection and belongingness among patients. Additionally, feedback mechanisms help professionals to monitor the detailed progress timely and design the individual training. The accountability-related measures consisted of the core part of the feedback mechanism, including physical data delivery [29,32,35], texts warning [10,34], active-reported intervention diary [10,28], online progress monitoring [34,42], regular consultation with professionals [38], and so on. The broader implication is that the effectiveness of human support components in these trials may be substantially underestimated relative to their potential, given how rarely participants accessed them as intended.

#### Motivational and Behavioral Strategies

A recurring finding was the gap between initial intention and long-term behavior, while motivational strategies addressed with only partial success. Across several included studies, self-efficacy was discussed as a potentially important factor in shaping adherence [26,27,42]. Studies in which participants received early clinical feedback (weight loss and improved biomarkers) reported higher engagement, a pattern consistent with the theoretical notion that observable, self-attributed progress may reinforce continued participation. Conversely, where such feedback was absent or delayed, motivation relied on external structures that proved fragile once formal supervision withdrew [34]. Additionally, some studies noted the spontaneous formation of peer networks through trusted social media platforms, such as Facebook (Meta) and LINE (LY Corporation), which appeared to provide a channel for sharing experiences and mutual support [10]. Notably, 2 studies [40,42] highlighted disease-specific health literacy as a potentially important enabling factor, given that patients who did not understand the reversibility of MASLD through lifestyle change lacked the foundational rationale for long-term engagement. This represents a modifiable barrier that precedes and conditions the effectiveness of all other motivational strategies.

#### Patient-Level Characteristics

Patient-level findings challenged several assumptions prevalent in digital health trial design. The association between older age and higher completion contradicts common concerns about technology adoption barriers and suggests that motivation and perceived necessity may outweigh digital literacy deficits when adequate onboarding is provided [34]. More striking is the paradox surrounding disease severity: greater comorbidity burden was in some cases associated with higher engagement [25,40], implying that clinical urgency may function as a motivational resource rather than a contraindication to digital delivery. In contrast, mental health comorbidities consistently attenuated adherence, revealing that behavioral monitoring alone cannot address, pointing to the need for integrated psychological support within lifestyle intervention frameworks [30]. Structural barriers related to occupation, insurance, and technology access further constrained participation in ways that reflect health inequities that digital delivery reduces but does not eliminate, particularly for women and racialized minority populations carrying disproportionate community and family obligations [34].

## Discussion

### Summary of Principal Findings

This mixed methods systematic review provides a comprehensive picture of attrition dynamics in digital MASLD interventions. Quantitatively, pooled retention across meta-analysis was 80%, with substantial between-study heterogeneity that no single participant-level moderator—mean age, BMI, sex distribution—was able to meaningfully explain. Neither intervention duration nor digital delivery modality significantly differentiated retention outcomes, though mobile app–based platforms demonstrated the highest point estimate and lowest within-group heterogeneity (section S7 in Multimedia Appendix 1). Qualitatively, facilitators and barriers operated across 4 intersecting domains: platform and design, human support and professional integration, motivational and behavioral strategies, and patient-level characteristics. Taken together, multifaceted determinants of attrition in digital MASLD self-management cannot be adequately captured by single variation, requiring the integration of quantitative data with patient-centered narrative evidence.

### Retention Rate in Digital Intervention

The point estimate observed in this synthesis (80%, 95% CI 72%‐87%) nominally exceeds the retention rates reported across several digital chronic-disease contexts. However, a definitive comparison cannot be established due to the wide PI (95% PI 47%‐95%) and substantial heterogeneity (*I*²=73.6%). In the field-defining meta-analysis by Meyerowitz-Katz et al [15], the pooled dropout rate across 17 app-based chronic-disease studies was 43% (95% CI 29%‐57%), corresponding to retention of roughly 57%. In smartphone-delivered mental health trials, Linardon and Fuller-Tyszkiewicz [19] reported a short-term study-attrition rate of 24.1%, aligning more closely with the present estimates. Several features of the included MASLD studies may partly contribute to the relatively favorable point estimate: a predominance of structured RCT designs with active follow-up (8/15 studies) [10,31,36,37,39,41,43,44], intervention durations frequently aligned with research-protocol windows rather than open-ended use, and the concentration of dropout reasons in situational rather than motivational categories. Consequently, such high pooled retention is likely a context-specific average rather than a guaranteed outcome for subsequent MASLD digital interventions. Ultimately, attrition is not an anomaly, but an intrinsic, structurally heterogeneous feature of eHealth trials [14].

A cross-cohort analysis of more than 100,000 participants in remote digital studies presented attrition curves with displayed steep early decay [49], and panel analysis of mental-health app usage by Baumel et al [50] found median open-day windows under 1 month. A similar trajectory was also observed in our study, suggesting that the drivers of long-term adherence differ qualitatively from those of early dropout in digital MASLD interventions. Limited by a small sample of 15 studies and substantial intrastratum heterogeneity, our analysis cannot definitively isolate true duration effects from confounding factors. Further studies should focus on exploring the underlying mechanisms that drive these 2 distinct phases, rather than treating attrition as a linear decay.

### Methodological Heterogeneity in Adherence Reporting

A critical methodological gap in the current literature is the systematic conflation of retention (study completion) with adherence (intervention fidelity). As previously noted, adherence in eHealth research remains conceptually underdeveloped, frequently misoperationalized, and rarely anchored in a prespecified definition of “intended use” [12]. Compounding this issue, objective platform-derived attrition metrics and patient-reported data are rarely integrated within the same analytical framework. Despite the longstanding recommendation of the WHO-endorsed mERA reporting checklist [51], few studies report fidelity-to-intervention and exposure-to-content in parallel. However, a promising trend toward behavior-specific adherence measurement, particularly for dietary and exercise components, is emerging. This granularity is of high clinical significance, as component-level reporting empowers interventionists to pinpoint specific behavioral failures and tailor support accordingly. Notably, Zeng et al [52] introduced the Exercise and Diet Adherence Scale to quantitatively assess adherence to lifestyle interventions among patients with MASLD. This scale represents a promising structured approach for evaluating compliance. But none of the studies included in this review applied it, which may be due to the digital format of interventions and the time limitation.

### Facilitators and Barriers of Attrition Management in Digital Intervention of MASLD

While the inductively derived 4-domain structure aligns with broader frameworks for digital chronic-disease interventions [16,53], our synthesis emphasizes that general digital tools must be specifically calibrated to the MASLD population.

The MASLD population is, on average, older, more multimorbid, and more digitally heterogeneous than the populations in which platform comparisons have most often been conducted (eg, mental health and smoking cessation) [1,54]. This profile amplifies the early access barriers, such as extra download steps or nonintegrated links. Consequently, the superior engagement achieved through preexisting messaging ecosystems (eg, LINE and WeChat [Tencent Holdings Ltd]) indicates that architectural embeddedness is a crucial, transferable design principle for this population. To further address this early attrition, stepped-care approaches demonstrate broader potential by calibrating to early engagement signals to offer a structural response [55]. Indeed, adaptive escalation triggered by early dropout markers has been shown to successfully optimize engagement trajectories in broader digital health implementations [56]. Moreover, digital platforms provide a structurally destigmatizing environment where patients can share their experiences without the social costs that drive avoidance behaviors. This affordance is particularly critical given the high prevalence of self-blame and shame documented in populations with MASLD [57,58].

Aligned with previous evidence that self-efficacy, relevant knowledge, and social support constitute the principal motivational substrates for MASLD self-management [59,60]. In MASLD, hepatic steatosis is asymptomatic in early and intermediate stages, biochemical and elastographic improvements occur over months [61], and patients lack the sensory cues that would otherwise maintain motivation in the interval [62]. Digital monitoring of distal proxies (weight and step count) therefore carries a heavier motivational load in MASLD than in conditions with intrinsic biological feedback. Consistently, foundational distinction between autonomous motivation (grounded in personally internalized goals) and controlled motivation (driven by external pressure) by Ryan and Deci [63] predicts that only the former reliably maintains long-term behavior change. This prediction is extensively confirmed in the meta-analytic synthesis by Ng et al [64] across 184 health-behavior studies. Further studies should investigate specific digital design strategies that cultivate this autonomous motivation in long-term MASLD management.

This review underscores the critical influence of both intervention design and patient-related characteristics on adherence. For lifestyle interventions, participant acceptance and adherence are significantly higher when the program resonates with their personal habits and cultural environment. Although digital delivery removes 1 dimension of structural inequity, it does not resolve broadband access, device compatibility, or the disproportionate domestic and caring obligations that, as several included studies documented, fell more heavily on women and minoritized populations [34]. Evidence suggests that participants with higher levels of education and socioeconomic resources generally demonstrate stronger adherence, highlighting the interplay between personal context and the effectiveness of self-management interventions [60]. However, many included studies in this review are pilot trials or feasibility studies, lacking robust sample sizes, long-term follow-up, and the statistical power to draw definitive conclusions. Therefore, future research should focus on implementing and evaluating personalized lifestyle programs within larger, real-world populations, exploring the socioeconomic factors to adherence and access.

### Integration of Quantitative and Qualitative Findings

While the meta-regression did not identify any study-level moderator that statistically explained between-study heterogeneity (section S7 in Multimedia Appendix 1), the integrated mixed methods analysis nevertheless yielded informative convergence between the 2 strands of evidence. Among delivery modalities, mobile app–based interventions showed the highest pooled retention of 83% (95% CI 73%‐90%; *I*²=66.9%; τ²=0.310) and, notably, the lowest within-group heterogeneity, suggesting a relatively consistent participant response to this format. This quantitative signal aligns with qualitative themes in which participants repeatedly emphasized the convenience, accessibility, and personalization afforded by smartphone-based platforms (Table 4). We interpret the absence of statistical significance in the subgroup and meta-regression analyses with caution rather than as evidence of no effect. Moreover, 3 factors plausibly account for this discrepancy. First, the included studies were unevenly distributed across delivery modalities, leaving most subgroup comparisons statistically underpowered. Second, design features such as interactivity, feedback, and personalization operate as continuous, multidimensional constructs that are poorly captured by coarse study-level moderator coding and are more faithfully represented in qualitative narrative. Third, participant- and context-level determinants were inconsistently or insufficiently reported at the study level, precluding their inclusion in the meta-regression.

This review specifically interrogates attrition dynamics in digital self-management interventions for MASLD. Whereas previous systematic reviews have predominantly evaluated clinical outcomes [13,65] or analyzed retention and adherence in isolation [15,16], this review integrates these 2 dimensions using a convergent segregated design. Driven by the substantial heterogeneity of adherence data in current literature, this mixed methods approach functionally contextualizes objective pooled dropout rates with qualitative, patient-centered narratives of adherence. This approach yields a comprehensive picture of attrition dynamics, revealing that while retention compares favorably to adjacent fields, sustaining true adherence depends on resolving entry friction, ensuring accessible human support, and aligning feedback with the asymptomatic disease course [61,62]. Although the conclusions warrant cautious interpretation given the predominance of pilot and feasibility designs among included studies, these findings offer a preliminary framework for prioritizing engagement architecture in the design and evaluation of future digital MASLD interventions.

### Strengths and Implications

Primarily, few mixed methods systematic reviews have examined adherence and retention to digital self-management interventions for MASLD, and our study adds to this emerging body of evidence. The integration of both quantitative and qualitative evidence provided a more holistic understanding of attrition conditions in digital self-management interventions among patients with MASLD. These findings carry practical and methodological implications for researchers and clinicians alike. Long-term engagement in digital MASLD interventions depends on accessible professional support and contextual integration into routine clinical care, suggesting that implementation design deserves as much attention as technology selection. Future research should prioritize clear and comprehensive reporting of attrition across multiple dimensions, including reasons for dropout, timing of disengagement, and differential dropout rates across intervention components, alongside standardized adherence definitions and multifaceted engagement metrics capturing task completion and behavior-specific fidelity. Longitudinal designs incorporating mediating constructs, such as motivation and self-efficacy, would further clarify how study participation translates into meaningful behavioral change and clinical benefit.

### Limitations

Several limitations warrant consideration. First, the overall certainty of evidence was rated very low under the GRADE framework, reflecting design constraints intrinsic to the digital intervention literature. The transparent nature of digital tools precludes participant and provider blinding in most included studies, and a large number of observatory studies further limit causal attribution. Second, despite the breadth of prespecified moderators examined, none reached statistical significance in subgroup analyses or meta-regression. The constraints of uneven study distribution across delivery modalities, the multidimensional nature of design features, and context-level determinants limit the integration of quantitative and qualitative findings. Third, the operationalization of retention varied across included studies. Although all reported a proportion completing their designated end point, specific definitions, follow-up windows, and counting rules differed and were not always explicitly stated, introducing definitional heterogeneity (*I*²=73.6%) into the pooled estimate. Fourth, the wide prediction interval indicates that the pooled retention estimate cannot be reliably extrapolated to specific clinical implementations without consideration of local contextual factors. Fifth, adherence was reported in 18 studies among included studies [10,25-27,29-32,34-38,40-44], that the metrics varied widely (eg, logins, app sessions, days of use, and percentage of behavioral targets met), and that this prevented quantitative pooling for adherence. Finally, included studies were predominantly conducted in high-income or East Asian settings, limiting generalizability to low- and middle-income contexts and underrepresented cultural populations.

### Conclusion

This mixed methods systematic review found that the pooled retention estimate in digital MASLD interventions nominally compared favorably with broader digital health benchmarks, yet substantial unexplained heterogeneity and a wide PI limit the generalizability of this finding. No participant-level or intervention-level moderator significantly differentiated retention in meta-regression. The qualitative synthesis highlighted facilitators and barriers spanning platform and design, human support and professional integration, motivational and behavioral strategies, and patient-level characteristics. Retention and adherence were empirically distinct across included studies, and pooled retention estimates alone are insufficient to evaluate intervention fidelity. Furthermore, the inconsistent operationalization of adherence constrains meaningful cross-study comparisons. Addressing this lack of measurement standardization must be a priority for methodological harmonization in future trials. Ultimately, identifying for whom and under what conditions these interventions are most effective will require continued innovation in both trial design and implementation strategies.

## Supplementary material

10.2196/89124 Multimedia Appendix 1 Supplementary appendices S1-S12.

10.2196/89124 Checklist 1 PRISMA 2020 checklist.

## Abbreviations

GRADE: Grading of Recommendations Assessment, Development and Evaluation
MASLD: metabolic dysfunction-associated steatotic liver disease
PI: prediction interval
PRISMA: Preferred Reporting Items for Systematic Reviews and Meta-Analyses
PRISMA-S: Preferred Reporting Items for Systematic Reviews and Meta-Analyses literature search extension
RCT: randomized controlled trial
