# Supplementary material for: Attrition in Digital Self-Management Interventions for Patients With Metabolic Dysfunction Associated Steatotic Liver Disease (MASLD): Mixed Methods Systematic Review
Source: J Med Internet Res. 2026 Aug 10;28:e89124. doi: 10.2196/89124 (PMC13456677; doi:10.2196/89124)
Supplement: Supplementary file 2 [file jmir-v28-e89124-s001.docx]

# Supplementary Appendices

**Contents**

[Supplementary Appendices	1](#_Toc13981)

[S1. Search strategies	2](#_Toc22887)

[S2. General Dimensions Across Included Digital MASLD Interventions	5](#_Toc13482)

[S3. Interactivity Dimensions Across Included Digital MASLD Interventions	7](#_Toc7151)

[S4. Retention Data	18](#_Toc28451)

[S5. Quality Appraisal	18](#_Toc15857)

[Table S5.1 Quality Appraisal of Quantitative Randomized Controlled Trials Using the Mixed Methods Appraisal Tool (MMAT, 2018 version).	18](#_Toc3577)

[Table S5.2 Quality Appraisal of Quantitative Non-Randomized Studies Using the Mixed Methods Appraisal Tool (MMAT, 2018 version).	19](#_Toc26487)

[Table S5.3 Quality Appraisal of Mixed Methods Studies Using the Mixed Methods Appraisal Tool (MMAT, 2018 version).	20](#_Toc16745)

[S6: Qualitative Analysis Process and Audit Trail	21](#_Toc30402)

[S6.1 Epistemological Position and Analytic Approach	21](#_Toc27098)

[S6.2 Data Extraction and Unit of Analysis	21](#_Toc284)

[S6.3 Three-Stage Coding Process	21](#_Toc11237)

[S7 Subgroup Analyses & Meta-Regression Analyses	25](#_Toc21324)

[S7.1 Subgroup Analyses	26](#_Toc14724)

[S7.2 Meta-Regression Analyses	27](#_Toc13958)

[S8 Deviations From the Registered Protocol	27](#_Toc8623)

[S9. Funnel Plot & Egger’ s Regression Test	29](#_Toc9749)

[S10. Certainty of Evidence Sub-section	30](#_Toc16885)

[S11. Explanation of Excluded Studies	31](#_Toc17163)

[Table S11.1 Studies excluded from the previously included pool after refined application of eligibility criteria	31](#_Toc4567)

[Table S11.2 Sensitivity analysis: pooled retention before vs. after re-inclusion of the three previously meta-analysed studies	31](#_Toc32442)

[S12. PRISMA-S Checklist	32](#_Toc32231)

## S1. Search strategies

| **N/O** | **Database** | **Index and Keyword Terms** | **Results** |
| --- | --- | --- | --- |
| **1** | **PubMed**  (NLM) | **#1**  "digitalization"[Title/Abstract] OR "Digital Health"[Title/Abstract] OR Telemedicine[Title/Abstract] OR sensor*[Title/Abstract] OR "internet of things"[Title/Abstract] OR APP[Title/Abstract] OR application[Title/Abstract] OR "digital human*"[Title/Abstract] OR "Digital technolog*"[Title/Abstract] OR "digital treatment*"[Title/Abstract] OR Telenursing[Title/Abstract] OR Teleprehabilitation[Title/Abstract] OR Tele-prehabilitation[Title/Abstract] OR Telehealth[Title/Abstract] OR "tele monitor*"[Title/Abstract] OR telemonitor*[Title/Abstract] OR e-health[Title/Abstract] OR eHealth[Title/Abstract] OR mHealth[Title/Abstract] OR m-health[Title/Abstract] OR "mobile health"[Title/Abstract] OR "mobile technolog*"[Title/Abstract] OR "mobile application*"[Title/Abstract] OR "mobile intervention*"[Title/Abstract] OR smartwatch*[Title/Abstract] OR "smart watch*"[Title/Abstract] OR "mobile"[Title/Abstract] OR "cell phone"[Title/Abstract] OR "cellular phone"[Title/Abstract] OR smartphone[Title/Abstract] OR "smart phone*"[Title/Abstract] OR iphone[Title/Abstract] OR android[Title/Abstract] OR windows[Title/Abstract] OR iOS[Title/Abstract] OR portable[Title/Abstract] OR ipad*[Title/Abstract] OR tablet[Title/Abstract] OR phone[Title/Abstract] OR telephone[Title/Abstract] OR app[Title/Abstract] OR apps[Title/Abstract] OR website[Title/Abstract] OR online[Title/Abstract] OR SMS[Title/Abstract] OR Message[Title/Abstract] OR Email[Title/Abstract] OR software[Title/Abstract]  **#2**  "Artificial Intelligence"[Title/Abstract] OR "Intelligent System*"[Title/Abstract] OR "Machine Learning"[Title/Abstract] OR "deep learning"[Title/Abstract] OR "Vector Machine"[Title/Abstract] OR "bayesian network*"[Title/Abstract] OR "Expert system*"[Title/Abstract] OR "neural network*"[Title/Abstract] OR AI[Title/Abstract] OR "Computer Reasoning"[Title/Abstract] OR "Machine Intelligence"[Title/Abstract] OR "Computational Intelligence"[Title/Abstract] OR "Digital Intelligent"[Title/Abstract] OR AI[Title/Abstract] OR LLM[Title/Abstract] OR "large language model "[Title/Abstract] OR chatgpt[Title/Abstract] OR chatbot[Title/Abstract] OR deepseek[Title/Abstract] OR wearable[Title/Abstract] OR web[Title/Abstract] OR software[Title/Abstract] OR "digital technology"[Title/Abstract] OR "smart technology"[Title/Abstract] OR "smart device"[Title/Abstract] OR "smart wearable device"[Title/Abstract] OR "augmented reality"[Title/Abstract] OR "virtual reality"[Title/Abstract] OR VR[Title/Abstract] OR AR[Title/Abstract] OR Internet[Title/Abstract] OR LoT[Title/Abstract] OR "brain-computer interface"[Title/Abstract] OR "brain-machine interface"[Title/Abstract]  **#3：**#1 OR #2  **#4:** "Non-alcoholic Fatty Liver Disease"[Mesh]  **#5**  NAFLD[Title/Abstract] OR MAFLD[Title/Abstract] OR MASLD[Title/Abstract] OR "Non-alcoholic Fatty Liver Disease"[Title/Abstract] OR "Metabolic dysfunction-associated fatty liver disease"[Title/Abstract]  **#6：**#4 OR #5  **#7：**#3 AND #6  Search date: 17 April 2026  Limits: English language  Publication date from 1 January 2015 to 17 April 2026 | **3899** |
| **2** | **Web of Science**  (Clarivate) | **#1:** TS=("Artificial Intelligence" OR "Intelligent System*" OR "Machine Learning" OR "deep learning" OR "Vector Machine" OR "bayesian network*" OR "Expert system*" OR "neural network*" OR AI OR "Computer Reasoning" OR "Machine Intelligence" OR "Computational Intelligence" OR "Digital Intelligent" OR AI OR LLM OR “large language model ” OR chatgpt OR chatbot OR deepseek OR wearable OR web OR software OR “digital technology” OR “smart technology” OR “smart device” OR “smart wearable device” OR “augmented reality” OR “virtual reality” OR VR OR AR OR Internet OR LoT OR “brain-computer interface” OR “brain-machine interface”  **#2:** TS=("digitalization" OR "Digital Health" OR Telemedicine OR sensor* OR "internet of things" OR APP OR application OR "digital human*" OR "Digital technolog*" OR "digital treatment*" OR Telenursing OR Teleprehabilitation OR Tele-prehabilitation OR Telehealth OR "tele monitor*" OR telemonitor* OR e-health OR eHealth OR mHealth OR m-health OR "mobile health" OR "mobile technolog*" OR “mobile application*” OR “mobile intervention*” OR smartwatch* OR "smart watch*" OR "mobile" OR "cell phone" OR “cellular phone” OR smartphone OR “smart phone*” OR iphone OR android OR windows OR iOS OR portable OR ipad* OR tablet OR phone OR telephone OR app OR apps OR website OR online OR SMS OR Message OR Email OR software ))  **#3:** #1 OR #2  **#4:** TS=(NAFLD OR MAFLD OR MASLD OR "Non-alcoholic Fatty Liver Disease" OR "Metabolic dysfunction-associated fatty liver disease”)  **#5:** #3 AND #4  Search date: 17 April 2026  Language: English  Publication Years: 2015-2025 | **10,622** |
| **3** | **Embase**  (Elsevier, Embase.com) | **#1**  nafld:ti,ab,kw OR mafld:ti,ab,kw OR masld:ti,ab,kw OR 'non-alcoholic fatty liver disease':ti,ab,kw OR 'metabolic dysfunction-associated fatty liver disease':ti,ab,kw  **#2**  'artificial intelligence':ti,ab,kw OR 'intelligent system*':ti,ab,kw OR 'machine learning':ti,ab,kw OR 'deep learning':ti,ab,kw OR 'vector machine':ti,ab,kw OR 'bayesian network*':ti,ab,kw OR 'expert system*':ti,ab,kw OR 'neural network*':ti,ab,kw OR 'computer reasoning':ti,ab,kw OR 'machine intelligence':ti,ab,kw OR 'computational intelligence':ti,ab,kw OR 'digital intelligent':ti,ab,kw OR ai:ti,ab,kw OR llm:ti,ab,kw OR 'large language model':ti,ab,kw OR chatgpt:ti,ab,kw OR chatbot:ti,ab,kw OR deepseek:ti,ab,kw OR wearable:ti,ab,kw OR web:ti,ab,kw OR 'digital technology':ti,ab,kw OR 'smart technology':ti,ab,kw OR 'smart device':ti,ab,kw OR 'smart wearable device':ti,ab,kw OR 'augmented reality':ti,ab,kw OR 'virtual reality':ti,ab,kw OR vr:ti,ab,kw OR ar:ti,ab,kw OR internet:ti,ab,kw OR lot:ti,ab,kw OR 'brain-computer interface':ti,ab,kw OR 'brain-machine interface':ti,ab,kw OR 'digitalization':ti,ab,kw OR 'digital health':ti,ab,kw OR telemedicine:ti,ab,kw OR sensor*:ti,ab,kw OR 'internet of things':ti,ab,kw OR application:ti,ab,kw OR 'digital human*':ti,ab,kw OR 'digital technolog*':ti,ab,kw OR 'digital treatment*':ti,ab,kw OR telenursing:ti,ab,kw OR teleprehabilitation:ti,ab,kw OR 'tele prehabilitation':ti,ab,kw OR telehealth:ti,ab,kw OR 'tele monitor*':ti,ab,kw OR telemonitor*:ti,ab,kw OR 'e health':ti,ab,kw OR ehealth:ti,ab,kw OR mhealth:ti,ab,kw OR 'm health':ti,ab,kw OR 'mobile health':ti,ab,kw OR 'mobile technolog*':ti,ab,kw OR 'mobile application*':ti,ab,kw OR 'mobile intervention*':ti,ab,kw OR smartwatch*:ti,ab,kw OR 'smart watch*':ti,ab,kw OR 'mobile':ti,ab,kw OR 'cell phone':ti,ab,kw OR 'cellular phone':ti,ab,kw OR smartphone:ti,ab,kw OR 'smart phone*':ti,ab,kw OR iphone:ti,ab,kw OR android:ti,ab,kw OR windows:ti,ab,kw OR ios:ti,ab,kw OR portable:ti,ab,kw OR ipad*:ti,ab,kw OR tablet:ti,ab,kw OR phone:ti,ab,kw OR telephone:ti,ab,kw OR app:ti,ab,kw OR apps:ti,ab,kw OR website:ti,ab,kw OR online:ti,ab,kw OR sms:ti,ab,kw OR message:ti,ab,kw OR email:ti,ab,kw OR software:ti,ab,kw  **#3:** #1 AND #2  Search date: 17 April 2026  Limit timespan=2015-2025  Language: English | **6072** |
| **4** | **Cochrane Library**  (Wiley) | **#1**  NAFLD OR MAFLD OR MASLD OR Non-alcoholic Fatty Liver Disease OR Metabolic dysfunction-associated fatty liver disease in Title Abstract Keyword  **#2**  "Artificial Intelligence" OR "Intelligent System*" OR "Machine Learning" OR "deep learning" OR "Vector Machine" OR "bayesian network*" OR "Expert system*" OR "neural network*" OR AI OR "Computer Reasoning" OR "Machine Intelligence" OR "Computational Intelligence" OR "Digital Intelligent" OR AI OR LLM OR “large language model ” OR chatgpt OR chatbot OR deepseek OR wearable OR web OR software OR “digital technology” OR “smart technology” OR “smart device” OR “smart wearable device” OR “augmented reality” OR “virtual reality” OR VR OR AR OR Internet OR LoT OR “brain-computer interface” OR “brain-machine interface” OR "digitalization" OR "Digital Health" OR Telemedicine OR sensor* OR "internet of things" OR APP OR application OR "digital human*" OR "Digital technolog*" OR "digital treatment*" OR Telenursing OR Teleprehabilitation OR Tele-prehabilitation OR Telehealth OR "tele monitor*" OR telemonitor* OR e-health OR eHealth OR mHealth OR m-health OR "mobile health" OR "mobile technolog*" OR “mobile application*” OR “mobile intervention*” OR smartwatch* OR "smart watch*" OR "mobile" OR "cell phone" OR “cellular phone” OR smartphone OR “smart phone*” OR iphone OR android OR windows OR iOS OR portable OR ipad* OR tablet OR phone OR telephone OR app OR apps OR website OR online OR SMS OR Message OR Email OR software in Title Abstract Keyword  **#3:** #1 AND #2  Search date: 17 April 2026  Limit timespan=2015-2025  Language: English | **5297** |
| **5** | **CINHAL**  (EBSCOhost) | **#1**  XB (NAFLD OR MAFLD OR MASLD OR "Non-alcoholic Fatty Liver Disease" OR "Metabolic dysfunction-associated fatty liver disease”)  **#2**  XB ("Artificial Intelligence" OR "Intelligent System*" OR "Machine Learning" OR "deep learning" OR "Vector Machine" OR "bayesian network*" OR "Expert system*" OR "neural network*" OR AI OR "Computer Reasoning" OR "Machine Intelligence" OR "Computational Intelligence" OR "Digital Intelligent" OR AI OR LLM OR “large language model ” OR chatgpt OR chatbot OR deepseek OR wearable OR web OR software OR “digital technology” OR “smart technology” OR “smart device” OR “smart wearable device” OR “augmented reality” OR “virtual reality” OR VR OR AR OR Internet OR LoT OR “brain-computer interface” OR “brain-machine interface” OR "digitalization" OR "Digital Health" OR Telemedicine OR sensor* OR "internet of things" OR APP OR application OR "digital human*" OR "Digital technolog*" OR "digital treatment*" OR Telenursing OR Teleprehabilitation OR Tele-prehabilitation OR Telehealth OR "tele monitor*" OR telemonitor* OR e-health OR eHealth OR mHealth OR m-health OR "mobile health" OR "mobile technolog*" OR “mobile application*” OR “mobile intervention*” OR smartwatch* OR "smart watch*" OR "mobile" OR "cell phone" OR “cellular phone” OR smartphone OR “smart phone*” OR iphone OR android OR windows OR iOS OR portable OR ipad* OR tablet OR phone OR telephone OR app OR apps OR website OR online OR SMS OR Message OR Email OR software)  **#3:** #1 AND #2  Search date: 17 April 2026  Filters applied: in the last 10 years, English. | **923** |

## S2. General Dimensions Across Included Digital MASLD Interventions

| **Project** | **First Author & Year** | **Country** | **Journal** | **Study Design** | **Sample Size (enrolled)** | **Mean Age**  **(year)** | **Male Distribution**  **%(n/total)** | **Mean BMI**  **(kg/m²)** | **Intervention Duration** |
| --- | --- | --- | --- | --- | --- | --- | --- | --- | --- |
| SMART-Liver | Kwon 2024 | Korea | Journal of Medical Internet Research | RCT | 138 | 48.9 | 61.7% (63/102) | NR | 24 weeks |
| Dr. Coach | Cho 2024 | Korea | Nutrients | RCT (pilot) | 60 | 41.4 | 94% (46/49) | 29.7 | 4 weeks |
| nBuddy/Nutritionist Buddy, Verita Analytics | Lim 2020 | Singapore | JMIR mHealth and uHealth | RCT | 108 | NR | NR | NR | 24 weeks |
| VITALISE | Hallsworth 2021 | U.K. | Journal of Medical Internet Research | Feasibility Study | 35 | 54 | 69% (24/35) | NR | 24 weeks |
|  | Avery 2025 |  | BMJ Open Gastroenterology | Mixed methods |  |  |  |  |  |
| Sidekick | Björnsdottir 2025 | Iceland | JMIR Cardio | Feasibility study | 38 | 59.5* | 0 | 37.6 | 12 weeks |
|  | Björnsdottir 2025 |  | JMIR Cardio | Long-term follow-up |  |  |  |  |  |
| Fitbit | Tincopa 2022 | U.S. | Digestive Diseases and Sciences | Mixed methods | 40 | 52.5 | 52.2% (21/40) | 33.9 | 24 weeks |
| Activity monitor (Forerunner 35, Garmin, Garching, Germany) | Haufe 2021 | Germany | Clinical and Translational Gastroenterology | RCT | 314 | 48.3 | NR | 33.6 | 24 weeks |
| BRIDGE | Dalal 2024 | U.S. | BMC public health | Mixed methods | 57 | 57* | 33% (19/57) | 31.9 | 12 weeks |
| Tele-ProEx | Freer 2024 | Australia | BMJ Open | Mixed methods | 28 | 63.5 | 7% (2/28) | NR | 12 weeks |
|  | Freer 2025 |  | The British Journal of Nutrition | RCT |  |  |  |  |  |
| NUTRIEASE | Liu 2024 | China | Nutrition Journal | RCT | 226 | 36.7 | 62.7% (106/169) | 29.2 | 12 weeks |
| Web-based exercise platform | Pfirrmann 2019 | Germany | Alimentary Pharmacology & Therapeutics | Prospective Study | 44 | 41* | 68.2%(30/44) | 31.3 | 8 weeks |
|  | Huber 2019 |  | Journal of Medical Internet Research |  |  |  |  |  |  |
| Web-based lifestyle modification program(developed on a Cloud/SaaS e-learning platform by Docebo SpA ) | Mazzotti 2018 | Italy | Journal of Hepatology | observatory | 716 | 46.0 | NR | 33.7 | 24 weeks |
|  | Petroni 2024 |  | Alimentary Pharmacology & Therapeutics | Long-term (5-year) follow-up |  |  |  |  |  |
| CareMessage | Axley 2018 | U.S. | Liver International | RCT | 30 | 52 | 33% (10/30) | 40 | 24 weeks |
| WeChat mini-program | Sun 2026 | China | Journal of Medical Internet Research | RCT | 89 | 40 | 61.8% (55/89) | 28.6 | 24 weeks |
| LINE Official Account | Kaewdech 2024 | Thailand | Scientific Reports | RCT | 122 | 53* | 35.2% (43/122) | 28* | 24 weeks |
| MyTatva RESET Care Program | Soni 2025 | India | World Journal of Hepatology | Observatory | 22 | 45.6 | 63.6% (14/22) | NR | 12 weeks |

Note. * median; NR: not reported.

## S3. Interactivity Dimensions Across Included Digital MASLD Interventions

| **Project** | **Anchor Paper** | **Digital Platform** | **Intervention Components** | **Interactive Touchpoints** | **Interaction Modality** | **Personalised** | **Contact Frequency** | **Interactivity Level** |
| --- | --- | --- | --- | --- | --- | --- | --- | --- |
| SMART-Liver | Kwon 2024 | Smartphone app | Dietary logging (AI-powered food photo recognition); weight and BMI self-monitoring; calorie tracking; exercise logging (type, duration, habits); NAFLD educational slides + diet news + exercise video clips; summary quizzes; personalised coaching sessions; weekly tailored feedback via app chat; automated daily SMS reminders (8 AM); monthly feedback reports from dietitian and physical activity specialist; goal-setting meetings (week 1, week 12) | Asynchronous in-app chat messaging with dietitian and physical activity specialist (tailored weekly feedback on diet logs); automated daily SMS missions (8 AM); real-time in-app chat for queries (responded during announced working hours); monthly feedback reports with dietitian comments; remote meeting counselling for goal-setting (week 1) and goal-reset (week 12/month 3) | Asynchronous in-app chat messaging + automated SMS (unidirectional daily missions) + synchronous remote video meeting (goal-setting sessions at months 1 and 3) | Yes — feedback tailored to individual logs and weight trajectory | Daily automated SMS (8 AM); weekly tailored feedback from research team on diet log performance rate; monthly feedback report from dietitian and physical activity specialist; real-time chat responses during announced working hours; remote counselling meetings at weeks 1 and 12 | ● HIGH |
| Dr. Coach | Cho 2024 | Smartphone app | 4-Week Intervention:  (1) Knowledge Education: MASLD definition, disease course, prognosis, nutrition, and exercise guidelines. (2) Goal Setting and Planning: SMART goals. (3) Self-Monitoring: Weight, daily diet, exercise, sleep, mood, along with feedback. (4) Action Assistance: Diet/activity reminders, alternative options, habit formation. (5) Meal Replacement: Concurrently combined with high-protein partial meal replacement (dinner replacement, 150 kcal per serving + low-fat milk/soy milk for a total of 240 kcal, containing approx. 25 g protein per serving). | Chat-format mobile dietitian coaching (5 days/week); self-monitoring feedback; weight/diet/exercise/sleep/mood logging; reminders. | Asynchronous chat messages (dietitian-led, 5 days/week) + automated reminders | Yes — Personalized nutrition prescriptions based on EMR data, taking 30 underlying diseases into consideration; individualized goal setting; personalized diet/exercise feedback. | SDietitian chat guidance 5 days a week; daily automated reminders; throughout the 4-week study period. | ● HIGH |
| nBuddy/Nutritionist Buddy, Verita Analytics | Lim 2020 | Smartphone app | 6-month intervention: Dietary diary logging system (including individualized calorie goals based on age/gender/physical activity level); automatic step tracking (synced with phone's built-in pedometer, goals increase weekly, reaching 10,000 steps by week 3); weight logging; video viewing function (weekly educational short videos); automated food choice evaluation system (generates lists of healthier, culturally appropriate alternatives); daily prompts and scheduled automated reminders (log diet daily, log weight twice weekly); daily/weekly/monthly chart reports; peer support chat channel (connects with family/friends); dietitian dashboard monitoring (real-time viewing of diet, exercise, and weight data to provide feedback and encouragement). | Dietitian monitors via dashboard in real-time and provides feedback and encouragement; automated food evaluation system (instant feedback); weekly educational videos; peer support chat channel; scheduled automated reminders. | Asynchronous dietitian feedback (dashboard monitoring) + automated algorithmic feedback (food evaluation) + one-way content (weekly videos, daily prompts) + peer chat (among participants). | Yes—individualized calorie goals based on user age/gender/physical activity level; individualized step goals based on pedometer baseline; dietitian provides personalized real-time feedback and encouragement; automated food evaluation provides culturally appropriate alternatives. | Continuous real-time dashboard monitoring by dietitian (frequency not explicitly specified but available in real-time); automated reminders daily (diet) and twice weekly (weight); step goals automatically increase weekly. | ● HIGH |
| VITALISE | Hallsworth 2021 (development & pilot) | Web app  (Changing Health platform; accessible on all devices) | 8 structured modules (NAFLD education, food, physical activity, self-regulation tools); goal setting; food/step/weight logging; lifestyle coach telephone support | Telephone coaching: initial session + weekly 10-min sessions over 12 weeks (Step 5 implementation plan); coaching accessed by only 3/11 participants in pilot | Synchronous telephone (coaching); self-directed web app (modules + self-monitoring tools) | Partial — coaching personalised to individual dietary/activity goals; module content standardised for all | Weekly coaching available for 12 weeks (pilot); continuous self-access to modules | ● HIGH |
|  | Avery 2025 (clinical feasibility) | Web app  (accessible on desktop, laptop, tablet, or smartphone) | MASLD-specific education modules; self-regulation tools (goal setting, food monitoring/logging, step tracking, weight monitoring); optional telephone lifestyle coaching | Monthly telephone coaching (~20 min initial; ~10 min follow-up); optional — only 8/35 patients attended ≥1 session (mean 4, range 1–6); coach initiates scheduled appointments | Synchronous telephone (coaching); self-directed web app (education + self-monitoring) | Partial — coaching personalised to individual goals and progress; educational content standardised | Monthly telephone coaching (optional); continuous self-access; mean 7 logins over 6 months (SD 11, median 3) | ● HIGH |
| Sidekick | Björnsdottir 2025 (Feasibility study) & Björnsdottir 2025 (long-term) | Smartphone app | 12-week intervention, daily missions: NAFLD/NASH disease education short videos + brief message content; meal photo logging (≥3 times/week) + meal health rating (sliding scale) + hunger/fullness assessment; manual step logging (individualized goal, based on week 1 baseline); mindfulness/meditation exercises (twice weekly, starting week 3); stress/sleep/energy level logging (2 days/week, 10-point VAS scale); bi-weekly surveys (food-related behaviors, NAFLD/NASH symptoms); motivation/knowledge/attitude surveys at weeks 1-2 and 11-12; weekly dietary goal: gradually reduce carbohydrates (individualized setting at week 2, based on week 1 intake). | Real human health coach (non-AI) asynchronous in-app messages: weekly feedback on food diaries and other in-app activities; participants can ask questions anytime (replied within 24 hours, excluding weekends); short web interviews provided in the first 2-3 weeks (to build connection, explain the program, discuss strengths and barriers). | Asynchronous in-app messaging (real health coach <-> participant) + automated daily missions (unidirectional). | Yes—coach provides personalized weekly feedback based on individual food diaries and in-app activities; carbohydrate reduction goals are individualized based on week 1 intake; step goals are individualized based on week 1 baseline. | Weekly coach feedback (guaranteed); participants can ask questions anytime (replied within 24 hours, excluding weekends); coaches sent an average of 23.5 messages to participants (SD 10.3), participants sent an average of 15.5 messages to coaches (SD 12.4), average response time 1.2 days (SD 0.9). | ● HIGH |
| Fitbit | Tincopa 2022 | Fitbit Zip wearable activity tracker (step monitoring) + email feedback system + 3-month telephone nutrition assessment | 6-month intervention: (1) Continuous Fitbit Zip wear (daily waking hours); (2) Individualized step goals (increased by 10% weekly, max increase of 800 steps/week, capped at 10,000 steps/week); (3) Individualized step feedback + motivational messages (via email, weekly for the first 3 months, bi-weekly for the next 3 months); (4) Baseline 24-hour dietary recall (conducted by researchers); (5) Telephone nutrition assessment at month 3 (reviewing the first 3 months' baseline results, providing overall physical activity and nutrition feedback); (6) 6-month follow-up visit (repeat anthropometrics, lab tests, Fibroscan, 6-minute walk test, PROMIS questionnaires, semi-structured exit interview); (7) Additional telephone/email contact for those with low step counts or consecutive missing data (≤ weekly). | Emails: Individualized step feedback + motivational messages (weekly for first 3 months; bi-weekly for next 3 months); month 3 telephone nutrition assessment (1 time, reviewing performance over the past 3 months); Fitbit data retrieved and analyzed weekly (by researchers); extra email/phone contact for participants with low compliance (≤ weekly); semi-structured exit interview (at month 6). | Asynchronous email feedback (primary modality) + one-time synchronous telephone nutrition assessment (month 3) + asynchronous phone/email contact when necessary (for low compliance). | Yes—step goals are individualized with a 10% weekly increase based on personal baseline; email feedback is tailored based on personal step performance; month 3 nutrition assessment is based on individual baseline dietary recall results. | Step feedback + motivational emails: weekly for the first 3 months, bi-weekly for the following 3 months; month 3 telephone nutrition assessment: 1 time; extra contact for low compliance: ≤ weekly (as needed); Fitbit data retrieved and analyzed weekly. | ● HIGH |
|  | Haufe 2021 | Telemonitoring: wearable activity tracker (Garmin Forerunner 35) + nutrition diary + custom health app — NOT video telehealth | 6-month exercise intervention; activity monitor worn continuously; 7-day food diary analysed by dietitians; personal counselling session at baseline; custom app for nutrition/stress/exercise info | Single baseline personal counselling session with exercise scientist; dietitian review of food diary; individualised feedback on training via online platform; NO synchronous video sessions | Asynchronous data upload + expert feedback via online platform; one-time in-person counselling | Yes — exercise prescription and feedback individualised based on HR, steps, and patient feedback | Continuous monitoring; regular expert feedback (frequency NR precisely); dietitian review once | ● HIGH |
| BRIDGE | Dalal 2024 | Group telehealth via videoconferencing (Zoom)—Shared Medical Appointments (SMA) format, led by Advanced Practice Providers (APPs: hepatology nurse practitioners + physician assistants), in an academic outpatient hepatology clinic setting (UCSF). | 6 SMA sessions (bi-weekly, approx. 90 minutes each): (1) 6 thematic psychoeducational lectures ("Intro to BRIDGE and MASLD", "Improving MASLD Health Outcomes", "Motivation and Change", "Dietary Habits for a Healthy Liver", "Physical Activity for a Healthy Liver", "Stress Management"); (2) Interactive group discussions/activities (determining readiness for behavior change, self-monitoring habits, plans for overcoming challenges); (3) Motivational Interviewing (MI) throughout; (4) Goal tracker (personal behavior change goal setting and tracking); (5) 5-minute 1-on-1 breakout rooms at the end of each session (each participant speaks individually with an APP); (6) Slides and educational materials distributed before each session; (7) Peer support—participants share experiences and support each other. | Synchronous group video conferences (Zoom): 15-20 minutes APP lecture + Q&A + 45-60 minutes group activity discussion; 5-minute 1-on-1 breakout room at the end of each session (APP and each participant individually); unstructured opening dialogue at the start of each session ("what is going well / what are the challenges?"); educational materials and slides distributed before sessions (unidirectional); medical assistant telephone support for registration and connection issues. | Synchronous group video consultations (Zoom SMA format) + synchronous 1-on-1 breakout room videos + unidirectional pre-session material distribution. | Partial personalization—1-on-1 breakout rooms provide personalized goal discussions; goal trackers set individualized behavior change goals; group discussions are based on participants' own situations (readiness for behavior change is highly individualized); educational content is standardized for all participants. | Bi-weekly for a total of 6 sessions (approx. 12 weeks), about 90 minutes each; educational materials distributed before sessions; no daily contact between sessions (no app, no message pushes, no daily monitoring). | ● HIGH |
| Tele-ProEx | Freer 2024 (RCT feasibility) & Freer 2025 (mixed methods) | Multi-platform telehealth: (1) TeleHab exercise prescription platform and app (VALD Health, Brisbane, Australia)—embedded exercise videos + tracking; (2) Zoom video conferencing ; (3) SMS; (4) Email; (5) Research Food Diary app (24-hour dietary recall). | 12-week Tele-ProEx multi-component intervention: (1) Individualized home strength training (prescribed by a certified Exercise Physiologist - EP), 3 times/week, approx. 25 minutes each, 6-8 exercises, 2 sets x 10-12 reps, moderate intensity (RPE 5-8); prescription updated at week 6; additionally requires 2 times/week aerobic training (≥60 mins/week); (2) Dietary intervention—dietitian designs a high-protein (1.2-1.5 g/kg/day) modified heart-healthy Mediterranean diet predominantly based on plant protein (legumes, tofu, nuts, seeds); (3) Behavioral support—3-4 SMS per week (exercise and plant protein intake health tips + motivational support); (4) Online protein checklist (weeks 2, 6, 9, 12, to monitor adherence to protein recommendations). | Zoom consultation in week 1 (EP exercise prescription + dietitian dietary plan education); Zoom video group consultations in weeks 2 and 6 (approx. 2-3 participants): EP monitors exercise progress + dietitian feedback on protein checklist; TeleHab app: embedded exercise videos + tracking (used by participants after each workout); 3-4 behavior change support SMS per week (unidirectional, automated); resource materials sent via email (meal plans, recipes, etc.); complete online protein checklist at weeks 2, 6, 9, 12. | Synchronous Zoom video (EP and dietitian consultations at weeks 1, 2, 6) + asynchronous TeleHab app (exercise tracking, autonomous participant use) + unidirectional SMS (behavioral support, 3-4 messages/week) + unidirectional email (resource materials). | Yes—exercise prescription is fully individualized by the EP based on personal conditions (based on fitness and daily routine); prescription updated at week 6; protein targets are determined based on age and gender; SMS content consists of generic health tips (non-individualized); dietary resources (recipes, meal plans) are generic. | Scheduled synchronous contact: Week 1 Zoom (EP + dietitian, 1 each), Week 2 Zoom (EP + dietitian group counseling), Week 6 Zoom (EP + dietitian group counseling)—total of 6 scheduled video consultations (over 12 weeks); continuous asynchronous: 3-4 SMS per week; autonomous on-demand: TeleHab app and protein checklist (weeks 2, 6, 9, 12). | ● HIGH |
| NUTRIEASE | Liu 2024 (comprehensive lifestyle — multicenter RCT, China) | Smartphone app (NUTRIEASE 8.9.12, Notte, China) + in-person clinical monitoring — NOT a standalone web platform | ILI group: low-carbohydrate (20–25%), high-protein (40–45%) calorie-restricted diet (25 kcal/kg×0.7); 2 daily nutrition replacement bars; food photo logging (3 days/4 weeks) via NUTRIEASE app; exercise: ≥150 min/wk moderate aerobic + 2×/wk resistance; 1-on-1 dietitian health education; weekly mobile counselling (≥15 min telephone/app); periodic science articles via app. Control group: balanced CRD (45–55% carbohydrate); standard counselling at weeks 4, 8, 12 only. | ILI group: weekly telephone or app message counselling with dietitian (≥15 min each); dietitian review of food photo logs every 4 weeks with online feedback; science articles pushed via app; scheduled follow-ups at weeks 4, 8, 12. Control group: scheduled follow-ups at weeks 4, 8, 12 only (standard counselling). | Synchronous telephone counselling + asynchronous app messaging (ILI group); unidirectional app content (science articles); asynchronous food photo log review with feedback | Yes (ILI group) — diet plan individualised at baseline; weekly counselling adjusts diet and PA plans to individual progress; 1-on-1 health management plan developed per participant obesity stage | ILI group: weekly telephone/app counselling (≥15 min) + food log review every 4 weeks + clinic visits at weeks 4, 8, 12. Control group: clinic visits at weeks 4, 8, 12 only. | ● HIGH |
| Web-based exercise platform | Pfirrmann 2019 (web exercise — intervention study) | Web-based individualised exercise platform with online feedback | 8 weeks of individualized endurance + strength training (Weeks 1-4: 3 times/week—endurance x2 + strength x1; Weeks 5-8: 5 times/week—endurance x3 + strength x2); website provides text/image + video instructions for strength movements; heart rate monitor (Polar FT1) + resistance bands provided; website has built-in exercise schedule, relaxation/breathing exercises; discussion board + chat room (peer support); weekly progress report filled by patients including average heart rate, resting heart rate, training time, and Borg scale uploaded; bi-weekly optional offline group training; 12-week continuation follow-up after the 8-week intervention. | Receive exercise recommendations via internal site messaging weekly; patients submit completed progress reports (average HR, resting HR, training duration, Borg scale, traffic light signals) every weekend -> exercise therapist replies and adjusts the next week's plan within 24 hours; reminders sent to those who fail to submit on time (average 2.8 reminders/person); standard email can be used as an alternative to website submission for feedback (165 times total); group training every two weeks. | Asynchronous in-platform messages (delivering exercise recommendations) + asynchronous email feedback (as an alternative) + bi-weekly offline/synchronous group training. | Yes - Individualized heart rate intensity developed based on baseline exercise testing (cardiopulmonary exercise testing, lactate measurement); dynamically adjusted weekly based on Borg scale + heart rate + personal feedback; traffic light principle uses pain scores as the primary decision-making basis. | Weekly exercise recommendations (in-site messaging); replies within 24 hours; 8-week active supervision period; weeks 8-20 follow-up period (no active plan adjustments); bi-weekly optional group training. | ● HIGH |
|  | Huber 2019 (web exercise — clinical biomarkers, Aliment Pharmacol Ther) | Same web-based exercise platform as Pfirrmann 2019 | 8-week individualised endurance + strength exercise (3–5×/wk); weekly plan adjustments; online feedback; NO dietary restrictions | Online platform: participant reports training duration, HR, exhaustion → sports medicine expert adjusts plan; individual counselling on exercise; recommendation to continue post week 8 | Asynchronous online bidirectional feedback (platform-mediated); individual expert counselling | Yes — 'individual training plan developed based on baseline exams with weekly adjustments based on participant's weekly feedback' | Weekly plan adjustments during 8-week active phase; then unsupervised continued access | ● HIGH |
| Web-based lifestyle modification program(developed on a Cloud/SaaS e-learning platform by Docebo SpA ) | Mazzotti 2018 & Petroni 2024 | Web-based intervention based on cloud/SaaS e-learning platform (Developed by Docebo SpA, Biassono, MB, Italy); login with username and password; accessible on any device; not a dedicated mobile app. | 4 educational modules (corresponding to offline GBI content): ① Energy balance, nutrients and weight monitoring; ② Dietary pyramid and portion sizes; ③ Food shopping and labels; ④ Physical activity (time and amount); + 5th module: Role-playing game (testing adherence to previous learning objectives); each module contains an introductory online questionnaire (awareness/motivation/competence) -> 25-35 slides with voiceover explanations and interactive exercises; slides are highly interactive, including fill-in-the-blank exercises and learning objective minigames; modules can be repeated infinitely; patients can submit food diaries or ask questions to the center offline (via a dedicated tool); face-to-face follow-up every 6 months (throughout the 2 years). | Asynchronous email contact (patient submits food diary or questions -> center replies); dedicated online submission tool; face-to-face follow-up clinic visits every 6 months (providing reinforcement and comorbidity management); system tracks module completion; most interactions occurred via email and were not systematically recorded. | Self-directed online module learning (unidirectional, educational content) + asynchronous email (bidirectional, questions/diary submission and reply) + synchronous face-to-face follow-up clinic visits every 6 months. | Partial - core module content is standardized; individualized enrollment assessment conducted via motivational interviewing (face-to-face meeting before enrollment); individualized reinforcement provided during follow-ups; email replies are partially individualized. | Continuous autonomous access to web modules (no time limit); email contact as needed; face-to-face follow-ups every 6 months (months 6, 12, 24); system tracks module completion. | ● HIGH |
| CareMessage | Axley 2018 (text messaging RCT) | Text messaging (SMS) — CareMessage platform | 22-week text messaging programme: 3 messages/week covering NAFLD education, nutrition, exercise, stress management, goal setting, behaviour change, overcoming barriers | Mixed uni- and bidirectional SMS: outgoing health messages (unidirectional) + 2-way question-and-response messages (bidirectional); participants reply to prompted questions about barriers and goals | Asynchronous SMS — mixed unidirectional (broadcast education) and bidirectional (prompted Q&A) | Partial — messages adapted based on participant replies to barrier questions; core content standardised; motivated by individual goal established early in programme | 3 messages/week for 22 weeks; response rate declined from 70% (week 1) to 41–45% (months 2–6) | ● HIGH |
| WeChat mini-program | Sun 2026 | WeChat mini-program (TLC program, Therapeutic Lifestyle Changes; jointly developed by the Departments of Gastroenterology, Sports Rehabilitation, and Clinical Nutrition at Xinhua Hospital Affiliated to Shanghai Jiao Tong University School of Medicine; no extra download needed, relies on WeChat ecosystem). | 4 modules, 24 weeks: ① Individualized diet and exercise plan formulation (based on baseline weight, metabolic disease status, and fatty liver severity; weight loss goal 5%-10%); ② Dietary intervention plan (program calculates daily caloric needs + recommended menus; food exchange portion replacements; daily photo uploads of food diaries -> dietitian analyzes and provides feedback via WeChat); ③ Exercise video courses (heart rate armband HW702 YESOUL for real-time recording; 30 mins per session; ≥5 times/week; alternative exercise forms can be selected and durations recorded); ④ Monitoring and informational support (daily check-ins; research team sends personalized feedback weekly based on task completion; smart push of educational articles). | Daily patient check-ins (diet + exercise task completion); daily food photo uploads with WeChat feedback from dietitian; research team sends personalized feedback messages weekly (based on task completion status); system smartly pushes educational articles (based on personal interests and focus areas); heart rate armband data automatically uploaded to the program; face-to-face clinic assessments at baseline and 6 months. | Asynchronous WeChat messages (patient <-> dietitian food diary feedback; bidirectional) + asynchronous WeChat personalized weekly feedback (research team -> patient; unidirectional) + automated push of educational articles (system -> patient; unidirectional) + automatic heart rate data upload (patient device -> system; unidirectional). | Yes - Diet plan is individualized based on baseline weight and metabolic status calculation; exercise goals are set individually; dietitian provides feedback specific to personal food diaries; weekly feedback is based on personal task completion; educational articles pushed based on personal interests. | Daily check-ins + food photo uploads; daily automated heart rate recording (during exercise); weekly personalized feedback from the research team; real-time/asynchronous dietitian food diary feedback (frequency tracks with upload frequency); daily educational article pushes; face-to-face clinic visits at weeks 0 and 24. | ● HIGH |
| LINE Official Account | Kaewdech 2024 | Social media messaging app (LINE Official Account; LINE application, Thailand) | 24-week intervention: standard LSI advice delivered by a single hepatologist at enrollment (dietary, physical activity, and exercise guidance via video clip — provided to both groups as SOC); intervention group additionally received infographics, video clips, and text content about MASLD disease education and LSI (diet, physical activity, exercise) broadcast to followers every 3–7 days via the LINE Official Account; content was curated by investigators, validated by hepatology specialists, and adhered to AASLD, EASL, APASL, and RCPT guidelines; patients could send messages back to the administrator (unidirectional broadcasting + optional bidirectional messaging); control group retained access only to the enrollment video clip via their own LINE Official Account (no active broadcasts or reminders). | Periodic broadcast of infographics, video clips, and text (every 3–7 days, unidirectional); patients could message back to administrator (administrator sees all; followers cannot see each other); no active broadcasts or reminders sent to control group. | Asynchronous broadcast messaging (unidirectional, primarily) + optional asynchronous participant-to-administrator messaging | No — content broadcasts were standardised for the entire intervention group; no individualised feedback reported | Broadcasts every 3–7 days for 24 weeks; follow-up clinic visits at weeks 12 and 24 | LOW |
| MyTatva RESET Care Program | Soni 2025 | Smartphone app (MyTatva digital health application) + IoT devices (body composition analyzer machine + smartwatch) | 12-week (90-day) intervention across three arms: Group A — personalized diet plan (Individual Dietary Intervention Strategy [IDIS] plan; gradual, sustainable dietary changes; introductory call from dedicated diet coach; 15-day interval monitoring and follow-up calls; food diary adherence tracking); Group B — personalized diet + structured exercise plan (FITT-principle exercise prescription tailored to physical activity level, job demands, and physical limitations; 15-day interval monitoring; progressive increase in intensity and duration); Group C — comprehensive RESET care (diet + exercise + cognitive behavioral therapy [CBT]); CBT sessions address psychological barriers [stress, dysregulated eating, sedentary lifestyle]; coping strategies, mindfulness, relaxation exercises, cognitive restructuring; continuous psychological support throughout. IoT devices (BCA machine and smartwatch) used by all groups for automated, continuous anthropometric monitoring. | Introductory onboarding call from diet/health/CBT coach (group-specific); 15-day interval follow-up calls and app check-ins (Groups A and B); continuous CBT session support (Group C); automated BCA data collection (IoT, passive/continuous); coach adjusts plans based on adherence and progress; app-based food diary and compliance monitoring. | Synchronous telephone/call (onboarding + follow-up coaching) + asynchronous app-based monitoring (food diary, BCA auto-fetch) + IoT automated data capture (smartwatch + BCA) | Yes — diet plans individualised using IDIS based on cultural dietary habits, lifestyle, and local food availability; exercise prescription based on physical activity level, job demands, and limitations; CBT sessions tailored to individual psychological barriers; all plans adjusted at 15-day intervals based on individual progress | Onboarding call at baseline; 15-day interval follow-up calls and app monitoring throughout 12 weeks; continuous IoT monitoring (daily, automated); final assessment at 90 days | HIGH |

## S4. Retention Data

| **Study** | **Total** | **Event** | **Design** | **Intervention** | **Type, Duration** | **Mean_Age** | **Gender Distribution** | **Mean_BMI** |
| --- | --- | --- | --- | --- | --- | --- | --- | --- |
| Liu 2024 | 111 | 79 | RCT | APP | Short-term, ≤12 weeks | 36.0 | 0.59 | 29.5 |
| Kwon 2024 | 51 | 48 | RCT | APP | Long-term, ≥24weeks | 51 | 0.71 | 29.6 |
| Kaewdech 2024 | 61 | 51 | RCT | text messaging | Long-term, ≥24weeks | 52.5 | 0.377 | 71.9 |
| Cho 2024 | 30 | 25 | RCT | APP | Short-term, ≤12 weeks | 23 | 1 | 30.04 |
| Lim 2020 | 55 | 50 | RCT | APP | Long-term, ≥24weeks | 46.8 | 0.58 | 30.1 |
| Axley 2018 | 13 | 5 | RCT | text messaging | Long-term, ≥24weeks | 54 | 0.15 | 39 |
| Sun 2026 | 45 | 37 | RCT | APP | Long-term, ≥24weeks | 39.3 | 0.533 | 29 |
| Haufe 2021 | 160 | 132 | RCT | telehealth | Long-term, ≥24weeks | 48.3 | 0.85 | 33.6 |
| Avery 2025 | 35 | 23 | Observatory study | APP | Long-term, ≥24weeks | 54 | 0.69 | NA |
| Björnsdottir 2025 | 38 | 34 | Observatory study | APP | Short-term, ≤12 weeks | 58.1 | 0.39 | 37.6 |
| Freer 2025 | 14 | 12 | Observatory study | telehealth | Short-term, ≤12 weeks | 63.2 | 0.143 | 33.7 |
| Mazzotti 2018 | 278 | 211 | Observatory study | web | Long-term, ≥24weeks | 46 | 0.669 | 33.7 |
| Dalal 2024 | 57 | 33 | Observatory study | telehealth | Short-term, ≤12 weeks | 57.3 | 0.33 | 31.9 |
| Tincopa 2022 | 40 | 33 | Observatory study | APP | Long-term, ≥24weeks | 50.9 | 0.525 | 33.6 |
| Huber 2019 | 44 | 43 | Observatory study | web | Short-term, ≤12 weeks | 42.1 | 0.659 | 30.8 |

Note. Data of mean age, gender distribution, and mean BMI are extracted from intervention group in studies; Median data were adjusted from the five-number summary by Luo 2018. (*D. Luo, X. Wan, J. Liu and T. Tong (2018), "Optimally estimating the sample mean from the sample size, median, mid-range and/or mid-quartile range", Statistical Methods in Medical Research, 27: 1785-1805.)*

## S5. Quality Appraisal

### Table S5.1 Quality Appraisal of Quantitative Randomized Controlled Trials Using the Mixed Methods Appraisal Tool (MMAT, 2018 version).

| **Author** | **Q1** | **Q2** | **Q3** | **Q4** | **Q5** | **Q6** | **Q7** | **Rate** | **Quality** |
| --- | --- | --- | --- | --- | --- | --- | --- | --- | --- |
| Liu 2024 | yes | yes | yes | yes | yes | yes | yes | **100%** | **High** |
| Freer 2025 | yes | yes | yes | yes | yes | no | yes | **85.7%** | **High** |
| Kaewdech 2024 | yes | yes | yes | yes | yes | yes | yes | **100%** | **High** |
| Lim 2020 | yes | yes | yes | yes | yes | yes | yes | **100%** | **High** |
| Kwon 2024 | yes | yes | yes | yes | yes | no | yes | **85.7%** | **High** |
| Haufe 2021 | yes | yes | yes | yes | yes | yes | yes | **100%** | **High** |
| Axley 2018 | yes | yes | yes | yes | yes | no | yes | **85.7%** | **High** |
| Cho 2024 | yes | yes | yes | yes | yes | no | yes | **85.7%** | **High** |
| Sun 2026 | yes | yes | yes | yes | yes | no | yes | **85.7%** | **High** |

*Studies were categorized as high (80–100%), moderate (40–79%), or low (0–39%) quality.

Q1~Q7:

1. Are there clear research questions?
2. Do the collected data allow to address the research questions?
3. Is randomization appropriately performed?
4. Are the groups comparable at baseline?
5. Are there complete outcome data?
6. Are outcome assessors blinded to the intervention provided?
7. Did the participants adhere to the assigned intervention?

### Table S5.2 Quality Appraisal of Quantitative Non-Randomized Studies Using the Mixed Methods Appraisal Tool (MMAT, 2018 version).

| **Author** | **Q1** | **Q2** | **Q3** | **Q4** | **Q5** | **Q6** | **Q7** | **Rate** | **Quality** |
| --- | --- | --- | --- | --- | --- | --- | --- | --- | --- |
| Mazzotti 2018 | yes | yes | can't tell | yes | yes | no | yes | **71.4%** | **Moderate** |
| Soni 2025 | yes | yes | yes | yes | yes | no | yes | **85.7%** | **High** |
| Huber 2019 | yes | yes | yes | yes | yes | no | yes | **85.7%** | **High** |
| Petroni 2024 | yes | yes | yes | yes | yes | yes | yes | **100%** | **High** |
| Björnsdottir 2025 | yes | yes | yes | yes | yes | no | yes | **85.7%** | **High** |
| Pfirrmann 2019 | yes | yes | yes | yes | yes | no | yes | **85.7%** | **High** |
| Björnsdottir 2025 | yes | yes | yes | yes | yes | yes | yes | **100%** | **High** |

*Q1~Q7:

1. Are there clear research questions?
2. Do the collected data allow to address the research questions?
3. Are the participants representative of the target population?
4. Are measurements appropriate regarding both the outcome and intervention (or exposure)?
5. Are there complete outcome data?
6. Are the confounders accounted for in the design and analysis?
7. During the study period, is the intervention administered (or exposure occurred) as intended?

### Table S5.3 Quality Appraisal of Mixed Methods Studies Using the Mixed Methods Appraisal Tool (MMAT, 2018 version).

| **Author** | **Q1** | **Q2** | **Q3** | **Q4** | **Q5** | **Q6** | **Q7** | **Rate** | **Quality** |
| --- | --- | --- | --- | --- | --- | --- | --- | --- | --- |
| Freer 2024 | yes | yes | yes | yes | yes | yes | yes | **100%** | **High** |
| Dalal 2024 | yes | yes | yes | yes | no | yes | no | **71.4%** | **Moderate** |
| Hallsworth 2021 | yes | yes | yes | yes | yes | yes | yes | **100%** | **High** |
| Tincopa 2022 | yes | yes | yes | yes | yes | yes | no | **85.7%** | **High** |
| Freer 2025 | yes | yes | yes | yes | yes | yes | yes | **100%** | **High** |
| Dalal 2024 | yes | yes | yes | yes | no | yes | no | **71.4%** | **Moderate** |
| Avery 2025 | yes | yes | yes | yes | yes | yes | yes | **100%** | **High** |

*Studies were categorized as high (80–100%), moderate (40–79%), or low (0–39%) quality.

Q1~Q7:

1. Are there clear research questions?
2. Do the collected data allow to address the research questions?
3. Is there an adequate rationale for using a mixed methods design to address the research question?
4. Are the different components of the study effectively integrated to answer the research question?
5. Are the outputs of the integration of qualitative and quantitative components adequately interpreted?
6. Are divergences and inconsistencies between quantitative and qualitative results adequately addressed?
7. Do the different components of the study adhere to the quality criteria of each tradition of the methods involved?

## S6: Qualitative Analysis Process and Audit Trail

### S6.1 Epistemological Position and Analytic Approach

The qualitative synthesis was conducted using framework synthesis, a structured analytic method suited to reviews that integrate qualitative evidence from heterogeneous study designs toward an applied policy or clinical question. Unlike grounded theory or interpretive phenomenological analysis, framework synthesis does not aim to generate new theory from primary data; rather, it organises and interrogates existing findings — in this case, author-reported facilitator and barrier data — within an explicit analytical structure that remains visible and auditable throughout the process.

The analytic stance adopted was critically interpretive: extracted data were treated not merely as transparent descriptions of participant experience, but as author-mediated representations of that experience, shaped by study design, measurement focus, and disciplinary framing. This distinction informed how extracts were coded — with attention to what investigators chose to report, how they framed participant accounts, and where interpretive distance between participant voice and author summary was apparent.

### S6.2 Data Extraction and Unit of Analysis

The unit of analysis was the **meaningful extract**: any discrete passage in the results, discussion, or participant-reported findings sections of an included study that attributed a facilitating or inhibiting effect to a specific programme feature, participant characteristic, or contextual factor. Extracts varied in form across studies, including:

- Direct participant quotations (e.g., "nothing new for me to do" — Avery 2025)
- Author summaries of participant-reported experiences (e.g., investigator attribution of login failure to loss of confidence — Hallsworth 2021)
- Investigator interpretations of observed engagement patterns (e.g., structural barrier to coaching access — Avery 2025)
- Quantitatively-grounded qualitative observations (e.g., response rate decline interpreted as automation fatigue — Axley 2018)

Each extract was logged in a coding matrix alongside: study identifier, data type (participant quotation / author summary / investigator interpretation), direction (facilitator / barrier / ambivalent), and the behavioural domain to which it pertained (access, engagement, dietary adherence, exercise adherence, retention).

Extracts were included regardless of whether the original study was qualitative, quantitative, or mixed-methods, provided the extract itself carried qualitative interpretive content. Pure numerical findings without accompanying interpretive framing were retained in the adherence rate narrative synthesis but excluded from thematic coding.

### S6.3 Three-Stage Coding Process

Coding proceeded through three iterative stages. The transition between stages is documented below with worked examples drawn from the actual dataset.

#### Stage 1: Open Descriptive Coding

Each extract was assigned one or more **descriptive codes** that captured its surface content without imposing interpretive structure. Codes were phrased as noun-verb constructions describing the mechanism reported (e.g., login failure → disengagement; coach responsiveness → accountability). At this stage, no attempt was made to group or hierarchise codes; the goal was comprehensive, fine-grained coverage of the raw data.

**Worked Example — Avery 2025 (VITALISE feasibility study):**

| **Raw Extract** | **Data Type** | **Descriptive Code** | **Direction** |
| --- | --- | --- | --- |
| "Engagement dropped once participants had completed all available modules — 'nothing new for me to do'" | Participant quotation (author-reported) | Content Exhaustion → Disengagement | Barrier |
| Only 8/35 participants accessed ≥1 coaching session; investigators identified the requirement to complete all 8 modules first as a structural barrier | Investigator interpretation | Structural Prerequisite → Coaching Underutilisation | Barrier |
| Delivery at point of NAFLD diagnosis capitalised on heightened receptiveness | Investigator interpretation | Diagnostic Timing → Motivation | Facilitator |
| Multi-morbidity caused MASLD self-management to be deprioritised relative to other conditions | Author summary | Competing Health Demands → Deprioritisation | Barrier |
| General engagement decline after month 1 | Investigator observation | Temporal Decay → Sustained Engagement Failure | Barrier |

**Worked Example — Björnsdottir 2025a (Sidekick active phase):**

| **Raw Extract** | **Data Type** | **Descriptive Code** | **Direction** |
| --- | --- | --- | --- |
| Early web-based interviews in weeks 1–2 established rapport and explained programme goals | Author summary | Early Rapport → Sustained Engagement | Facilitator |
| Coach responsiveness within 24 hours explicitly cited as facilitator | Participant quotation (author-reported) | Coach Responsiveness → Accountability | Facilitator |
| 3/38 withdrew due to loss of interest; no re-engagement strategies were available | Investigator observation | loss of Interest → Unaddressed Dropout | Barrier |

**Worked Example — Freer 2024/2025 (Tele-ProEx):**

| **Raw Extract** | **Data Type** | **Descriptive Code** | **Direction** |
| --- | --- | --- | --- |
| 86% of participants had rarely consumed tofu or tempeh at baseline; cultural unfamiliarity substantially limited dietary adherence | Author summary | Cultural Unfamiliarity → Dietary non-adherence | Barrier |
| Inability to pause or rewind exercise videos cited as a specific barrier to exercise completion | Participant quotation (author-reported) | Platform Inflexibility → Exercise non-completion | Barrier |
| EP monitoring of RPE and pain after each session via TeleHab enabled rapid modification | Author summary | Real-time Monitoring → Safety and Adaptation | Facilitator |
| Only 14% of participants met both dietary targets simultaneously despite high exercise satisfaction | Investigator interpretation | Component Discordance → Selective Adherence | Barrier |

#### Stage 2: Focused Coding and Constant Comparison

Descriptive codes were progressively grouped through **constant comparison**: each new code was systematically compared with existing codes to identify conceptual similarities, distinctions, and hierarchical relationships. Where two codes described the same underlying mechanism in different studies, they were collapsed into a single focused code with multiple study anchors. Where superficially similar codes reflected meaningfully distinct mechanisms, they were retained as separate entries.

This stage produced the set of **focused codes** from which themes were subsequently derived. Key decisions made during this stage, and their rationale, are documented below.

**Decision log — selected examples:**

| **Decision** | **Rationale** |
| --- | --- |
| Login failure → disengagement (Hallsworth 2021) and primary access difficulty → dropout (Avery 2025) collapsed into single focused code: **entry barrier → durable disengagement** | Both extracts described the same mechanism: an access failure at programme entry producing disengagement not reversed by subsequent outreach. The shared mechanism justified collapsing despite surface terminological differences. |
| Coach responsiveness → accountability and scheduled coach contact → personal accountability retained as distinct focused codes | The first captured reactivity (response to participant-initiated contact); the second captured proactive structure (predetermined contact intervals). Although both pertained to coaching, they represented distinct accountability mechanisms with different design implications. |
| Content exhaustion → disengagement (Avery 2025) separated from content variety decline → maintenance dropout (Björnsdottir 2025b) | Content exhaustion referred to the absence of new material once modules were completed (a structural design ceiling); content variety decline referred to reduced engagement diversity after coaching ended (a temporal engagement dynamic). Different mechanisms warranted separate codes. |
| Cultural unfamiliarity → dietary non-adherence (Freer 2024/2025) assigned to Domain IV (Patient-Level: Cultural factors) rather than Domain I (Content structure) | Although the barrier manifested at the level of dietary content, its origin was participant-level cultural context rather than programme design. The coding decision followed the locus of the mechanism, not its surface presentation. |
| Automated reminder → habit maintenance and automated contact → declining response over time retained as separate codes under the same theme (1.3 Automation) | These represented the facilitating and barrier dimensions of the same intervention feature, capturing its time-varying effect. Retaining both codes preserved the ambivalent nature of automation as an engagement strategy. |

#### Stage 3: Theme Development and Domain Construction

Focused codes were grouped into **analytical themes** based on shared underlying mechanisms — not merely topical similarity. Themes were then organised into **domains** representing the level of the intervention system at which the mechanism primarily operated (platform, professional support, motivational, participant-level).

The transition from focused codes to themes involved a further layer of interpretive judgement: identifying what each cluster of codes revealed about engagement dynamics that individual codes did not. This analytical move is documented below for each domain.

**Domain I — Platform and Design Characteristics**

Three themes emerged from codes pertaining to technical and design features of the digital platform itself.

Theme 1.1 (Usability and Accessibility) grouped codes sharing the mechanism of **access friction**: features that increased or decreased the cognitive and technical effort required to enter and remain within the programme. The analytical insight at theme level — not visible at the individual code level — was that access failure at entry produced qualitatively different disengagement from content-level dropout: it was non-reversible in multiple studies, suggesting a threshold effect rather than a gradient.

Theme 1.2 (Content Structure) grouped codes pertaining to **information architecture**: how content was sequenced, formatted, and bounded. The analytical insight was the identification of content exhaustion as a distinct barrier type — structurally produced rather than motivationally driven — that had not been explicitly named as a construct in the primary literature.

Theme 1.3 (Automation) grouped codes pertaining to **algorithmic engagement features** operating without human mediation. The analytical insight was the temporal ambivalence of automation: a facilitator of habit formation early in the intervention trajectory that became progressively less effective over time, suggesting a ceiling on what automated contact can sustain independently of human support.

**Domain II — Human Support and Professional Integration**

Three themes emerged from codes pertaining to professionally mediated or peer-mediated support.

Theme 2.1 (Professional Coaching) grouped codes around the mechanism of **personalised accountability through expert contact**. The analytical insight — emerging from the juxtaposition of coaching value ratings among users with low uptake rates — was the structural paradox: coaching was positioned where it was least accessible to those who needed it most. This insight was not available from any single study but emerged from cross-study comparison.

Theme 2.2 (Group and Peer Support) grouped codes around **social accountability and stigma reduction through shared experience**. The analytical insight was the structural limitation of group-only formats: without inter-session digital contact, group-based accountability was episodic rather than continuous, creating engagement gaps between sessions.

Theme 2.3 (Personalised Feedback) grouped codes around **adaptive content delivery responsive to individual participant data**. The analytical insight was the distinction between bidirectional personalisation (enabling genuine adaptation) and one-way broadcast (delivering fixed content to all participants regardless of individual response), and the scalability tension that intensive personalisation creates.

**Domain III — Motivational and Behavioural Strategies**

Two themes emerged from codes pertaining to psychological and behavioural engagement mechanisms.

Theme 3.1 (Initial Motivation and Disease Context) grouped codes around the role of **perceived illness relevance and health literacy** in initiating engagement. The analytical insight was that disease-specific health literacy functioned as a prerequisite rather than a component: its absence attenuated the effectiveness of all subsequent motivational strategies.

Theme 3.2 (Goal Setting and Behavioural Monitoring) grouped codes around **structured self-regulation mechanisms** — including progressive goal calibration, motivational interviewing, and accountability tracking. The analytical insight was the post-intervention relapse pattern: behavioural gains sustained during active supervision were not maintained once accountability structures were withdrawn, suggesting that the mechanisms supporting adherence were extrinsic rather than internalised.

**Domain IV — Patient-Level Characteristics**

Three themes emerged from codes pertaining to characteristics participants brought to the intervention.

Theme 4.1 (Demographics and Clinical Profile) grouped codes around **individual-level clinical and demographic moderators of engagement**. The analytical insight was the counter-intuitive direction of several associations: older age and greater disease severity facilitated rather than inhibited engagement under conditions of adequate support, inverting assumptions common in digital health trial design.

Theme 4.2 (Socioeconomic and Structural Factors) grouped codes around **material and structural constraints on participation** that operated independently of motivation or design quality. The analytical insight was the distinction between barriers that telehealth delivery reduces (transportation) and barriers it does not resolve (device access, connectivity, insurance costs).

Theme 4.3 (Cultural and Dietary Factors) grouped codes around **cultural context as a determinant of dietary adherence and platform uptake**. The analytical insight was that cultural fit operated at two levels simultaneously: the content level (food recommendation acceptability) and the platform level (ecosystem familiarity), and that misalignment at either level could undermine adherence independently of motivation.

## S7 Subgroup Analyses & Meta-Regression Analyses

### S7.1 Subgroup Analyses

Results of all pre-specified subgroup analyses are summarised in Table S7.1. No statistically significant between-subgroup differences were detected for any of the three moderators examined.

***1. Study Design.***

The pooled retention proportion was 0.81 (95% CI: 0.67–0.90; I² = 73.7%; τ² = 0.499) among RCTs (k = 8) and 0.80 (95% CI: 0.61–0.90; I² = 73.8%; τ² = 0.539) among observational studies (k = 7). The between-subgroup difference was non-significant (χ²(df = 1) = 0.05, p = .820), indicating that study design did not account for the observed heterogeneity.

***2. Digital Delivery Modality.***

Among studies employing mobile applications (k = 8), the pooled retention rate was 0.83 (95% CI: 0.73–0.90; I² = 66.9%; τ² = 0.310), which was the highest point estimate across all delivery modes and was associated with the lowest within-group heterogeneity. Telehealth studies (k = 3) yielded a pooled estimate of 0.75 (95% CI: 0.30–0.96; I² = 85.7%; τ² = 0.516). For text messaging (k = 2) and web-based interventions (k = 2), the random-effects confidence intervals degenerated to [0.00, 1.00] due to insufficient studies for stable τ² estimation; the point estimates (0.65 and 0.91, respectively) should be interpreted with extreme caution and are presented for descriptive purposes only. The overall test for between-subgroup differences was non-significant (χ²(df = 3) = 1.78, p = .619).

***3. Intervention Duration.***

Short-term interventions (≤12 weeks; k = 6) yielded a pooled retention of 0.82 (95% CI: 0.59–0.93; I² = 76.6%; τ² = 0.743), and long-term interventions (≥24 weeks; k = 9) yielded 0.80 (95% CI: 0.68–0.89; I² = 71.3%; τ² = 0.427). The between-group test was non-significant (χ²(df = 1) = 0.04, p = .836). It is noted that the short-term subgroup showed greater within-group heterogeneity (τ² = 0.743) than the long-term subgroup (τ² = 0.427), which is reflected in the substantially wider HKSJ-adjusted confidence interval for the short-term estimate. This divergence in within-subgroup variance estimates, rather than a true duration effect, accounts for the apparently paradoxical pattern in which the fixed-effect model produced a borderline significant result (p ≈ .05) that disappeared entirely under the random-effects model.

**Table S7.1. Summary of subgroup analysis results.**

| **Subgroup Variable** | **k** | **Pooled Proportion [95% CI]** | **I² (%)** | **τ²** | **P (between-group)** |
| --- | --- | --- | --- | --- | --- |
| Study Design | | | | | |
| RCT | 8 | 0.81 [0.67, 0.90] | 73.7 | 0.4992 | 0.820 |
| Observational study | 7 | 0.80 [0.61, 0.90] | 73.8 | 0.5385 |  |
| Digital Delivery Mode | | | | | |
| Mobile application (APP) | 8 | 0.83 [0.73, 0.90] | 66.9 | 0.3104 | 0.619 |
| Text messaging | 2 | 0.65 [0.00, 1.00]ᵃ | 89.9 | 1.9811 |  |
| Telehealth | 3 | 0.75 [0.30, 0.96] | 85.7 | 0.5164 |  |
| Web-based | 2 | 0.91 [0.00, 1.00]ᵃ | 84.7 | 2.8951 |  |
| Intervention Duration | | | | | |
| Short-term (≤12 weeks) | 6 | 0.82 [0.59, 0.93] | 76.6 | 0.7426 | 0.836 |
| Long-term (≥24 weeks) | 9 | 0.80 [0.68, 0.89] | 71.3 | 0.4265 |  |

Note. k = number of studies; τ² = between-study variance; I² = percentage of total variation attributable to heterogeneity; P (between-group) = p value for the test of subgroup differences (χ²). ᵃ Confidence interval degenerated to [0.00, 1.00] due to insufficient studies (k = 2) for stable τ² estimation; results are descriptive only.

### S7.2 Meta-Regression Analyses

Three univariable meta-regression models were fitted on the logit scale using REML estimation with HKSJ-adjusted inference. None of the three continuous moderators examined—mean age, mean BMI, or proportion of male participants—was a statistically significant predictor of retention (all p > .20; Table S6.2). Furthermore, neither mean age (β = −0.012, 95% CI: −0.067 to 0.042, p = .628; R² = 0.00%) nor mean BMI (β = −0.003, 95% CI: −0.053 to 0.046, p = .882; R² = 0.00%) accounted for any of the observed between-study heterogeneity. The proportion of male participants showed a non-significant positive association with retention (β = 1.277, 95% CI: −0.902 to 3.456, p = .228) and explained 6.97% of the residual heterogeneity variance, though this should be interpreted cautiously given the absence of statistical significance. Substantial residual heterogeneity remained in all three models (residual I² = 80.2%–84.5%), indicating that the identified participant-level characteristics are insufficient to explain the observed variability in retention across studies.

**Table S7.2. Results of univariable meta-regression analyses for continuous participant-level moderators.**

| **Moderator** | **k** | **β (logit scale)** | **95% CI** | **P value** | **Residual τ²** | **R²** |
| --- | --- | --- | --- | --- | --- | --- |
| Mean age (years) | 15 | −0.0124 | [−0.067, 0.042] | .628 | 0.5136 | 0.00% |
| Mean BMI (kg/m²) | 14ᵃ | −0.0034 | [−0.053, 0.046] | .882 | 0.5631 | 0.00% |
| Male proportion (%) | 15 | 1.2771 | [−0.902, 3.456] | .228 | 0.4326 | 6.97% |

Note. β = regression coefficient on the logit scale (REML estimator, Hartung-Knapp-adjusted t-test); 95% CI = Wald-type confidence interval from the Knapp-Hartung procedure; R² = proportion of between-study variance explained by the moderator (truncated to 0% where the estimate is negative); Residual τ² = between-study variance remaining after accounting for the moderator. ᵃ One study excluded due to missing BMI (k = 14).

## S8 Deviations From the Registered Protocol

| **ID** | **Domain** | **Registered protocol** | **Actual implementation** | **Rationale** |
| --- | --- | --- | --- | --- |
| D1 | Disease nomenclature | Non-alcoholic fatty liver disease (NAFLD) | Metabolic dysfunction-associated steatotic liver disease (MASLD) | Updated per 2023 international Delphi consensus (Rinella et al., Hepatology, 2023). Population scope unchanged. |
| D2 | Separation of retention and adherence | Single composite outcome: 'factors influencing feasibility and adherence' | Retention and adherence treated as distinct outcomes: retention synthesised quantitatively (meta-analysis); adherence synthesised narratively | Data extraction revealed that these constructs were operationally distinct across studies. Separation improves conceptual precision and methodological transparency. |
| D3 | Intervention scope | Interventions implemented mainly by mobile apps (smartphone-focused) | All digital self-management platforms included: apps, web-based, telehealth, text messaging. | Pilot screening indicated that restricting to smartphone apps would exclude substantively relevant studies addressing the same clinical question. Broadening was determined prior to full-text review. |
| D4 | Statistical synthesis method | Risk ratio (RR) using random-effects model | Single-group proportion meta-analysis (logit transformation, HKSJ correction, REML) — no RR calculated | Seven of 15 included studies were single-arm designs without a comparator, rendering RR uncalculable for the full evidence base. Single-proportion meta-analysis is the methodologically appropriate approach for this data structure. |
| D5 | Retention data extraction rule | Latest available follow-up retention rate | Retention rate at end of active intervention period (not post-intervention follow-up) | Ensures comparability across studies with heterogeneous post-intervention follow-up designs. Sensitivity analysis (leave-one-out) examined the impact of this decision. |
| D6 | GRADE certainty assessment | Not specified | GRADE Summary of Findings table added for retention outcome | Added at explicit editorial request during peer review. Does not alter any pre-specified findings. |
| D7 | Search database coverage | CINAHL, Embase (Ovid), Embase.com, MEDLINE, PubMed | Above plus Web of Science, Cochrane Library; manual reference checking added; search updated | Additional databases ensure broader interdisciplinary coverage. Search updated to address editorial concern about currency. |

## S9. Funnel Plot & Egger’ s Regression Test

**Figure S9.1 Funnel Plot**

**Egger’ s Regression Test:**

**Linear regression test of funnel plot asymmetry:**

Test result: t = 1.50, df = 13, p-value = 0.1573

Bias estimate: 1.4954 (SE = 0.9963)

Details:

- multiplicative residual heterogeneity variance (tau^2 = 3.4777)

- predictor: standard error

- weight: inverse variance

- reference: Egger et al. (1997), BMJ

## S10. Certainty of Evidence Sub-section

**Table S10.1 summarises the certainty of evidence for the primary outcome of participant retention, assessed using the Grading of Recommendations, Assessment, Development and Evaluations (GRADE) framework. Certainty was evaluated across five domains: risk of bias, inconsistency, indirectness, imprecision, and small-study effects (publication bias). The starting certainty level was set at Low because the evidence base includes observational studies (single-arm designs without control groups). Downgrading decisions are explained in the footnotes below the table.**

| **Outcome** | **Studies (n)** | **Pooled Proportion (95% CI)** | **Prediction Interval** | **Risk of Bias** | **Inconsistency** | **Indirectness** | **Imprecision** | **Publication Bias** | **Certainty of Evidence** |
| --- | --- | --- | --- | --- | --- | --- | --- | --- | --- |
|  |  |  |  | **Assessment (direction)** | **Assessment (direction)** | **Assessment (direction)** | **Assessment (direction)** | **Assessment (direction)** | **Rating** |
| Retention rate in digital MASLD interventions | k = 15 (8 RCTs, 7 obs.) n = 1,032 | 80.38% (95% CI: 71.54%–86.98%) | 47.01%–94.98% (t-distribution, df = 14) | Serious ^a^ (↓1) | Serious ^b^ (↓1) I² = 73.6%; PI spans 47%–95%; unexplained by meta-regression | Not serious (–) PICO directly matched | Not serious (–) 95% CI acceptable; n = 1,032 | Not detected (–)  Egger p = 0.157; Begg p = 0.216 | ⊕◯◯◯ VERY LOW |

a	Risk of bias: Among the 8 RCTs, blinding of participants and intervention providers was not feasible given the nature of digital interventions, raising concerns in MMAT criteria Q2 and Q3. Observational studies (k = 7) were single-arm designs without control groups, limiting causal inference. Downgraded one level (Serious).

b	Inconsistency: Substantial between-study heterogeneity was detected (I² = 73.6%, 95% CI: 56.0%–84.2%; τ² = 0.4650; Q = 53.05, df = 14, p < 0.0001). The prediction interval (47.01%–94.98%) indicates that in a new comparable setting, true retention could plausibly range from below 50% to nearly 95%. Univariate meta-regression of age, BMI, and sex distribution did not explain this variability (all R² ≤ 6.97%). Downgraded one level (Serious).

c	Indirectness: The population (adults with MASLD), intervention (digital self-management tools), and outcome (participant retention) directly correspond to the research question. No meaningful indirectness identified. Not downgraded.

d	Imprecision: The 95% confidence interval (71.54%–86.98%) is acceptably narrow given the sample size (n = 1,032). Although the prediction interval is wide (reflecting true heterogeneity rather than imprecision per se), the pooled estimate itself is based on sufficient events (816/1,032). Not downgraded.

e	Small-study effects (publication bias): Egger's linear regression test showed no significant asymmetry (t = 1.50, df = 13, p = 0.157). Begg's rank correlation test was similarly non-significant (z = 1.24, p = 0.216). Not downgraded.

## S11. Explanation of Excluded Studies

### Table S11.1 Studies excluded from the previously included pool after refined application of eligibility criteria

| **Study (first author, year)** | **Reason category** | **Specific rationale** | **Contributed to original meta-analysis?** |
| --- | --- | --- | --- |
| Stine 2023 | C1 — Population | Cohort recruited under NASH histological criteria; cardiometabolic risk factors required by the 2023 MASLD definition not documented at the participant level. | Yes |
| Sato 2023 | C1 — Population | NASH-defined cohort without participant-level reporting of cardiometabolic comorbidity required for MASLD eligibility. | Yes |
| Alkhouri 2024 | C1 — Population | Predominantly NASH population in a prescription digital therapeutic trial; the MASLD-eligible subgroup could not be isolated from the reported data. | Yes |
| Dong 2016 | C2 — Intervention | Long-term follow-up of an offline lifestyle intervention; the digital component served only as a remote monitoring channel and did not constitute active digital self-management. | Yes |
| Motz 2021 | C1 — Population | NASH-defined population; criteria required by MASLD not reported. | No (narrative only) |
| Salmanizadeh 2025 | C3 — Outcomes | Qualitative interviews with gastroenterology/hepatology specialists; no patient-level engagement outcomes reported. | No (narrative only) |

Reason categories: C1 = Population (legacy NAFLD/NASH cohort not meeting the 2023 MASLD nomenclature criteria); C2 = Intervention (digital element used only for follow-up/monitoring, not as an active self-management component); C3 = Population/Outcomes (non-patient participants or absence of patient-level engagement outcomes). Re-screening was performed independently by two reviewers, blinded to study results, and completed before re-fitting of the meta-analyses.

### Table S11.2 Sensitivity analysis: pooled retention before vs. after re-inclusion of the three previously meta-analysed studies

| **Analysis** | **k** | **n** | **Events** | **Pooled retention** | **95% CI** | **95% PI** | **I²** | **τ²** |
| --- | --- | --- | --- | --- | --- | --- | --- | --- |
| **Main** (refined eligibility) | 15 | 1,032 | 816 | 80.4% | 71.5%–87.0% | 47.0%–95.0% | 73.6% | 0.465 |
| **Sensitivity** (Stine 2023 + Sato 2023 + Alkhouri 2024 + Dong 2016 re-included) | 19 | 1,235 | 1,001 | 82.5% | 75.1%–88.0% | 49.4%–95.8% | 75.6% | 0.523 |
| **Δ (sensitivity − main)** | +4 | +203 | +185 | **+2.1 pp** | wider lower bound, narrower upper bound | comparable | +2.0 pp | +0.058 |

Random-effects meta-analysis of proportions with logit transformation (PLOGIT), restricted maximum-likelihood (REML) estimation of τ², Hartung-Knapp-Sidik-Jonkman adjustment of the confidence and prediction intervals, and inverse-variance weighting. CI, confidence interval; PI, prediction interval; pp, percentage points.

Re-inclusion of the Four studies (Stine 2023, Sato 2023, Alkhouri 2024, Dong 2016) shifts the pooled retention estimate by approximately 2 percentage points upwards, which is well within the 95% confidence interval of the main analysis. The 95% prediction interval, between-study heterogeneity (I²), and τ² are essentially unchanged. The direction of the effect and the clinical interpretation — that retention in digital MASLD self-management interventions is high on average but varies substantially across settings — are robust to this analytic choice. This robustness supports the decision to base the primary synthesis on the refined eligibility while retaining the sensitivity analysis as a transparency safeguard.

## S12. PRISMA-S Checklist

| **SECTION/TOPIC** | **ITEM** | **CHECKLIST ITEM** | **DETAILS** |
| --- | --- | --- | --- |
| INFORMATION SOURCES AND METHODS | | |  |
| Database name | 1 | Name each individual database searched, stating the platform for each. | PubMed (NLM), Web of Science (Clarivate), Embase (Elsevier, Embase.com), Cochrane Library (Wiley), and CINAHL (EBSCOhost). (Methods, Search Strategy; Supplementary Appendix S1) |
| Multi-database searching | 2 | If databases were searched simultaneously on a single platform, state the name of the platform, listing all of the databases searched. | Not applicable, each database searched individually on its native platform. (Methods, Search Strategy) |
| Study registries | 3 | List any study registries searched. | No dedicated searches of clinical trial registries were undertaken. The Cochrane Library, including the Cochrane Central Register of Controlled Trials (CENTRAL), was searched as one of the bibliographic databases; however, this was not considered a separate search of study registries. |
| Online resources and browsing | 4 | Describe any online or print source purposefully searched or browsed (e.g., tables of contents, print conference proceedings, web sites), and how this was done. | None searched. |
| Citation searching | 5 | Indicate whether cited references or citing references were examined, and describe any methods used for locating cited/citing references (e.g., browsing reference lists, using a citation index, setting up email alerts for references citing included studies). | Reference lists of included studies were screened to identify additional records. (Methods, Search Strategy) |
| Contacts | 6 | Indicate whether additional studies or data were sought by contacting authors, experts, manufacturers, or others. | Not applicable. No authors or experts were contacted. |
| Other methods | 7 | Describe any additional information sources or search methods used. | None |
| SEARCH STRATEGIES | | |  |
| Full search strategies | 8 | Include the search strategies for each database and information source, copied and pasted exactly as run. | Full search strategies for all five databases are provided verbatim in Supplementary Appendix S1. |
| Limits and restrictions | 9 | Specify that no limits were used, or describe any limits or restrictions applied to a search (e.g., date or time period, language, study design) and provide justification for their use. | Limits applied: publication date 2015–2026 (last 10 years), English language. Justification: capture contemporary digital health interventions; team's working language. (Methods, Search Strategy; Supplementary Appendix S1) |
| Search filters | 10 | Indicate whether published search filters were used (as originally designed or modified), and if so, cite the filter(s) used. | No published/validated search filters were used. The strategy was developed by the review team. (Methods, Search Strategy) |
| Prior work | 11 | Indicate when search strategies from other literature reviews were adapted or reused for a substantive part or all of the search, citing the previous review(s). | No prior review's search strategy reused. |
| Updates | 12 | Report the methods used to update the search(es) (e.g., rerunning searches, email alerts). | The initial search was performed in June 2025 (yielding 22,864 citations) and was updated by rerunning the exact search strategies in April 2026 (yielding 3,949 new citations). (Results, Study Selection) |
| Dates of searches | 13 | For each search strategy, provide the date when the last search occurred. | All five databases were last searched on 17 April 2026. |
| PEER REVIEW | | |  |
| Peer review | 14 | Describe any search peer review process. | The search strategy was developed and reviewed iteratively by the review team; no formal external peer review was conducted. |
| MANAGING RECORDS | | |  |
| Total records | 15 | Document the total number of records identified from each database and other information sources. | The total number of records identified, including the breakdown per database, is detailed in Supplementary Appendix S1 and the PRISMA flowchart. |
| Deduplication | 16 | Describe the processes and any software used to deduplicate records from multiple database searches and other information sources. | Automated deduplication was performed using EndNote X20.5, followed by manual verification. (Methods, Selection Process) |
